# Trafficking Deficiency of TMEM175 Variants in Parkinson's Disease Pathogenesis and the Prospects of Precision Medicine

**DOI:** 10.1002/advs.76738

**Published:** 2026-07-31

**Authors:** Ting Luo, Yu He, Shuyao Li, Haoyu Guan, Ruili Cui, Yu Shi, Zhongwen Jiang, Siyu Wang, Xuan Li, Jiyuan Li, Mei Hu, Yupeng Zhou, Beisha Tang, Yanyan Zhang, Zhaobing Gao, Yu Zhou, Zhenhua Liu, Ping Li

**Affiliations:** ^1^ School of Pharmaceutical Sciences Guizhou Medical University Guiyang China; ^2^ School of Pharmaceutical Sciences Southern Medical University Guangzhou China; ^3^ Department of neurology & National Clinical Research Center for Geriatric Disorders Xiangya Hospital Central South University Changsha China; ^4^ Key Laboratory of Hunan Province in Neurodegenerative Disorders Xiangya Hospital Central South University Changsha China; ^5^ College of Pharmacy Nanjing University of Chinese Medicine Nanjing China; ^6^ School of Pharmacy Macau University of Science and Technology Taipa Macau China; ^7^ Zhongshan Institute for Drug Discovery Zhongshan China; ^8^ Shanghai Institute of Materia Medica Chinese Academy of Sciences Shanghai China; ^9^ Pharmacology Laboratory Zhongshan Hospital Guangzhou University of Chinese Medicine Zhongshan China; ^10^ School of Pharmacy Xinjiang Medical University Urumqi China

**Keywords:** agonist, intracellular trafficking, lysosomal ion channel, Parkinson's disease, TMEM175

## Abstract

The transmembrane protein 175 (TMEM175) is a high‐risk gene for Parkinson's disease (PD) and encodes an ion channel in lysosomes. Many PD‐associated variants in TMEM175 lead to the loss of ion channel function. In this study, we identified the PD‐associated TMEM175‐L156P variant is a missense mutation with trafficking deficiency. Using imaging, biochemical, and whole‐endolysosomal electrophysiological approaches, we identified that L156P abolished the lysosomal expression of TMEM175; instead, it accumulates in the endoplasmic reticulum (ER). The cytosolic segment of the fourth transmembrane helix (TM4‐1), where the L156 resides, determined the lysosomal localization of TMEM175. Having understood the structural basis, we evaluated wild‐type (WT) TMEM175 and pharmacological chaperones previously used in cystic fibrosis treatment. We elucidated that WT partially rescued the aberrant expression of L156P. Moreover, the chaperones substantially reduced the ER‐retained L156P and facilitated its trafficking to lysosomes. We further screened and identified a bifunctional chemical molecule that both corrected the trafficking defects and potentiated the ion channel function of L156P. Notably, the chemical‐restored L156P is functional, as it relieves the lysosomal over‐acidification. Conclusively, we establish the relationship between aberrant trafficking and pathogenesis in PD, demonstrating the potential of utilizing “chaperone plus agonist” bifunctional chemical molecules as personalized treatment for PD.

## Graphical abstract

This study identifies that the PD‐associated TMEM175‐L156P variant disrupts lysosomal ion channel trafficking by causing aberrant endoplasmic reticulum retention. A “chaperone–agonist” bifunctional small molecule restores TMEM175‐L156P lysosomal localization and channel function, thereby alleviating PD‐relevant cellular phenotypes and highlighting a genotype‐informed precision therapeutic strategy.

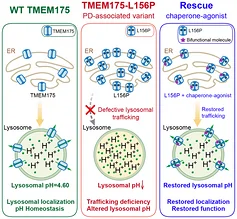

## 1 Introduction

Protein function depends heavily on the proper folding, assembly, trafficking, stability, and functionality in specific cellular compartments [1]. For membrane proteins in particular, such as ion channels and transporters, stringent quality control in the endoplasmic reticulum (ER) is required to ensure correct folding and assembly before they can enter the secretory pathway and ultimately reach their functional destinations, including the plasma membrane and lysosomes [2, 3]. Disruption of this process often leads to the retention of misfolded or improperly assembled proteins in the ER, a phenomenon known as trafficking deficiency [1]. This mechanism has been widely implicated in human diseases, especially in ion channel‐related disorders (channelopathies), where mutations impair protein trafficking rather than directly altering channel activity. A well‐known example is the F508del mutation in the cystic fibrosis transmembrane conductance regulator (CFTR‐*Δ*F508), which causes cystic fibrosis [4], as well as mutations in hERG and Nav1.5 channels associated with cardiac arrhythmias [5, 6, 7]. These studies establish trafficking deficiency as a unifying pathogenic mechanism in plasma membrane ion channels.

In parallel, lysosomal membranes also harbor a diverse set of ion channels and transporters that are essential for maintaining the lysosome homeostasis [2, 8]. Emerging evidence indicates that defects in the trafficking of lysosomal membrane proteins can similarly lead to severe diseases [9, 10, 11]. For instance, the mutations in NPC1 [10, 11] and cystinosin [9] result in their aberrant retention in the ER, causing the Niemann‐Pick disease type C (NPC) and cystinosis, respectively. Despite these advances, whether trafficking deficiency represents a general pathogenic mechanism for lysosomal ion channels remains insufficiently explored.

TMEM175 is one of the high‐risk factors linked to Parkinson's disease (PD) with an earlier age of onset [12, 13, 14, 15, 16]. Recently, transmembrane protein 175 (TMEM175) has been identified as a proton ion channel with the permeability to K^+^ and Cs^+^, targeted explicitly in lysosomes [15, 17, 18, 19, 20, 21, 22]. Human TMEM175 consists of 504 amino acids with a double leucine motif at the C‐terminus for potential lysosome targeting [8, 17, 20, 22, 23]. This channel operates as a homo‐dimer, composed of two repeats of six transmembrane domains (2 x 6TM) in each subunit, with TM1‐1 to TM6‐1 in the first repeat and TM1‐2 to TM6‐2 in the second repeat [20, 21, 22, 24]. Physiologically, the TMEM175 channel is the “acid gate” in the lysosome, allowing protons to be released from the lumen to the cytosol, which helps prevent over‐acidification in lysosomes [15, 17, 25]. Therefore, TMEM175 plays a fundamental role in various lysosomal functions, including maintaining lysosomal pH homeostasis [15, 23, 26], supporting proteolytic activity [13, 15, 27], and promoting autophagic flux [13, 23]. These functions contribute to effective degradation of alpha‐synuclein aggregates in the central nervous system [13, 14, 15, 26, 27, 28].

Dozens of TMEM175 variants, such as M393T, have been clinically identified in PD patients worldwide [12, 29, 30, 31, 32]. Recent pathological studies revealed that the first identified variant of TMEM175, M393T, functions as a loss‐of‐function variant on the lysosomal membrane [14, 15, 27, 28]. Additionally, Palomba et al. and Zhu et al. disclosed that several variants of TMEM175 in Italian patients significantly impaired the ion channel function, potentially contributing to the pathophysiology of PD [26, 30, 33]. Nevertheless, we screened TMEM175 variants associated with PD and identified six variants that are not directly involved in the ion channel function. However, the underlying mechanism remains unknown. These observations raise an important and unresolved question: could certain TMEM175 variants contribute to PD through defective intracellular trafficking rather than intrinsic functional impairment?

To address this possibility, we identified one of the six variants as a trafficking‐deficient variant, that is, a missense variant of TMEM175 which substitutes leucine with proline in position 156 (L156P). Through a careful investigation, we demonstrated that L156P is completely depleted from the lysosome and mis‐located to the ER instead. This mis‐localization leads to a loss of the physiological expression and ion channel function of TMEM175 on the lysosomal membrane. We discovered that the cytosolic segment in the transmembrane helix of TM4‐1, where L156 resides, plays a critical role in determining the lysosomal expression of TMEM175. Additionally, the homolog TM4‐2 in the second repeat to TM4‐1 in the first repeat, is also pivotal in assembling the correct channel in lysosomes. We further examined the subcellular expression of L156P with wild‐type (WT) TMEM175 to explore the potential pathogenesis in the heterozygous patient. As a dimeric channel, WT TMEM175 can partially, but not entirely, re‐locate L156P back to the lysosomal membranes. Interestingly, several small molecule chemical chaperones, particularly Corr‐4a, which is used for treating CFTR‐*Δ*F508, significantly reduced the accumulation of L156P in the ER and facilitated its trafficking to the lysosome. Importantly, the lysosome‐targeted L156P restored by Corr‐4a is functional as it relieves the lysosomal over‐acidification. We ultimately screened chemicals in a compound library and found that a synthetic agonist of the TMEM175 channel, DCY‐09‐X, can also effectively assist L156P in reaching the lysosomal membrane. In summary, our findings identify trafficking deficiency as a previously underappreciated pathogenic mechanism for TMEM175 variants in PD. By extending the concept of trafficking‐deficient mutations to lysosomal ion channels, our study not only advances our understanding of PD pathogenesis but also highlights intracellular trafficking as a potential therapeutic target for precision medicine of PD patients.

## 2 Results

### 2.1 A L156P Variant of TMEM175 is Identified in PD Patients

To test if any TMEM175 variants could be trafficking‐deficient, we conducted the genetic analysis by whole‐exome sequencing or whole‐genome sequencing in 3879 patients with PD from Parkinson's Disease & Movement Disorders Multicenter Database and Collaborative Network in China (PD‐MDCNC). Variants from TMEM175 were filtered by the same strategy used in our previous study [29]. The minor allele frequency (MAF) of variants was evaluated in the East‐Asian population using the gnomAD and ExAC databases, and variants with MAF < 0.001 were considered rare. Missense variants scored no less than 20 in Combined Annotation Dependent Depletion (CADD) values were predicted as damaging variants. Finally, we identified twenty‐one PD‐specific rare non‐synonymous variants for TMEM175, which contained 15 missense variants, 5 frame‐shifted variants, and 1 stop‐gained variants (Figure 1A). We generated a plasmid library by fusing variants with a N‐terminal EGFP tag. After transient over‐expression of these constructs and LAMP1‐mCherry, a biomarker of lysosomes, in COS1 cells, we explored the intracellular localization with a confocal microscope. It demonstrated that most missense variant were remained in lysosomes, and all frame‐shifted and stop‐gained variants were absent (Figure 1B, C; and Figure S1A–T). Interestingly, L156P (c.467T>C) in the fourth transmembrane helix (TM4‐1) is the only one missense variant that was depleted from lysosomes (Figure 1A–T). The TMEM175 p.L156P variant was observed at a low frequency in our cohort, with a minor allele frequency of approximately 6.32E‐04. Consistent with this, p.L156P is also rare in population reference datasets, with allele frequencies is 2.53E‐05 in East Asian populations reported by gnomAD. In our patient cohort, the TMEM175 p.L156P variant was identified in a total of three affected individuals originating from three unrelated families (Figure 1D).

**FIGURE 1:**
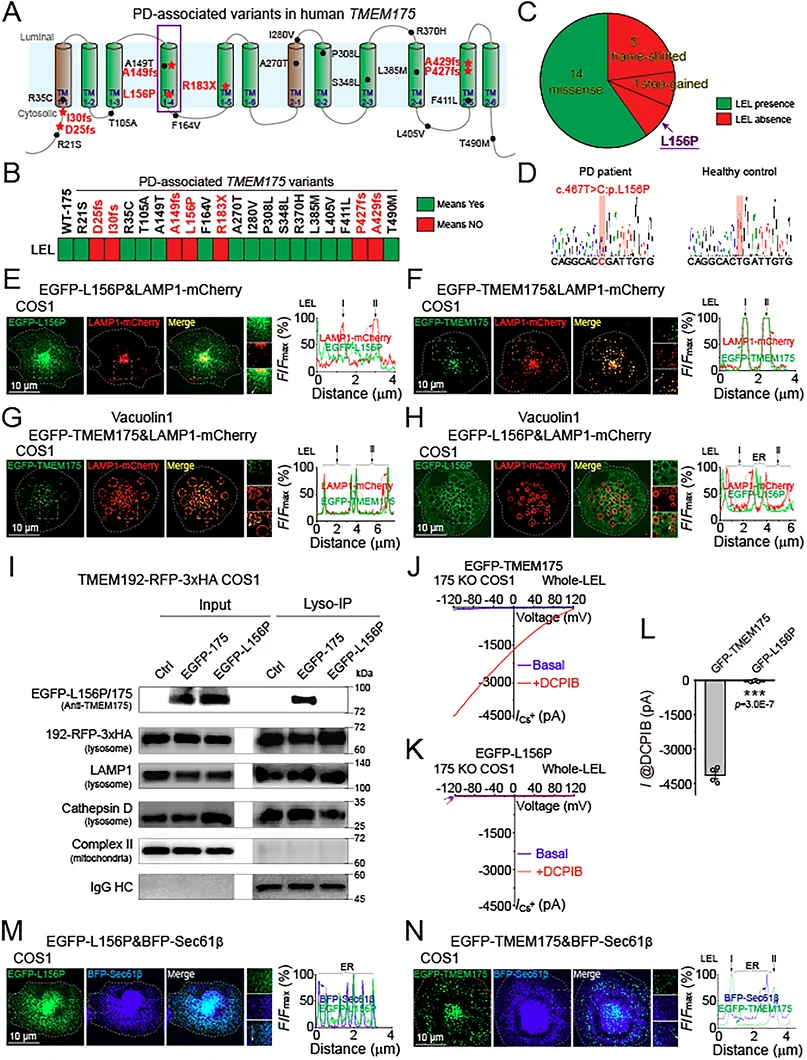
Identification of the PD‐associated hTMEM175‐L156P as a trafficking‐deficient variant. (A) Schematic representation of the topological structure of human TMEM175 protein and location of variants associated with PD. D25fs, I30fs, A149fs, L156P, R183X, P427fs and A429fs are labelled with stars in red. TM4‐1 is indicated by the purple box. (B) Summary of intracellular expression of various variants in human TMEM175. LEL: late endosome and lysosome. (C) Pie chart for lysosome expression of various variants in TMEM175. L156P is the only one missense variant impairing the lysosomal expression of TMEM175. (D) Sanger sequencing chromatograms show that a heterozygous missense variant (c. 467T>C: p. L156P) in the proband occurred. (E, F) Fluorescence microscopy shows the subcellular localization of EGFP‐L156P (E) and EGFP‐TMEM175 (F) in TMEM175 KO COS1 cells. EGFP‐L156P and EGFP‐TMEM175 are shown in green; lysosomes are labeled in Red (LAMP1‐mCherry). The graph to the Right of each panel shows the fluorescence intensity of a line scan (white arrows on the blown‐up image) through the double‐labeled object. Scale bar = 10 µm. n = 4. (G, H) Fluorescence microscopy shows the expression of EGFP‐TMEM175 (G) and EGFP‐L156P (H) in the enlarged lysosomes of TMEM175 KO COS1 cells. The membrane of enlarged lysosomes is labeled in Red (LAMP1‐mCherry). The graph to the Right of each group of images is a line scan through two separated lysosomes (LEL‐i and LEL‐ii) shown in the boxed region, indicating the intensity of EGFP‐TMEM175 or EGFP‐L156P (green lines) and LAMP1‐mCherry (red lines) along the white arrow. Cells were treated with vacuolin‐1 for 12–18h after transfection. Scale bar = 10 µm. n = 4. (I) Western blot from lysosomal fractions of COS1 cells stably expressing TMEM192‐mRFP‐3xHA after transfecting with EGFP‐TMEM175 or EGFP‐L156P for 24h. These fractions were immune‐isolated with magnetic beads coated with an anti‐HA antibody that recognizes the 3xHA epitope in TMEM192‐mRFP‐3xHA. n = 3. (J, K) Representative currents from lysosomal membranes of TMEM175 KO COS1 cells overexpressed with EGFP‐TMEM175 (J) and EGFP‐L156P (K) in the presence of DCPIB. The Cs+ currents recording paradigm on a lysosome enlarged by vacuolin‐1. The pipette solution is (in mM): 145 Cs+, 140 MSA^−^, 5 Cl^−^, 20 HEPES, pHL = 7.20; and the bath solution is: 145 NMDG+, 140 MSA^−^, 5Cl^−^, 20 HEPES, pHC = 7.20. A ramp protocol (−120 to +120 mV, 200 ms, holding at 0 mV) was used to record the current at 5 s intervals. (L) DCPIB potentiated Cs+ current amplitude of lysosomes from TMEM175 KO cells transfected with EGFP‐TMEM175 but not EGFP‐L156P. Data are mean ± s.e.m. from four independent experiments (n = 4). One‐way ANOVA. (M, N) Representative images show the subcellular localization of EGFP‐L156P (M) and EGFP‐TMEM175 (N) in TMEM175 KO COS1 cells. ER are labeled in blue (BFP‐Sec61b). The graph to the Right of each panel shows the fluorescence intensity of a line scan (white arrows on the blown‐up image) through the double‐labeled object. Scale bar = 10 µm. n = 4.

### 2.2 L156P Abrogates Lysosomal Expression of TMEM175 and Mis‐Localizes to the ER

To understand the molecular mechanism of L156P, we compared the intracellular expression pattern of EGFP‐L156P with EGFP‐TMEM175 in COS1 cells. Interestingly, EGFP‐L156P formed intracellular networks without any colocalization with lysosomes (Figure 1E), while EGFP‐TMEM175 was specifically presented in lysosomes (Figure 1F). As a membrane protein, EGFP‐TMEM175 was restricted to the membrane of lysosomes enlarged by a chemical, vacuolin‐1 (Figure 1G). In contrast, EGFP‐L156P did not appear to localize to the enlarged lysosomal membrane (Figure 1H). To further investigate the subcellular localization of EGFP‐L156P, we immunoprecipitated intact lysosomes using the well‐established Lyso‐IP method [9, 34] in COS1 cells stably expressing TMEM192‐mRFP‐3xHA. We observed significant enrichment of EGFP‐TMEM175 bands in COS1 cells overexpressing EGFP‐TMEM175, while there were negligible amounts of EGFP‐L156P bands in cells overexpressing EGFP‐L156P (Figure 1I). Furthermore, we evaluated the channel function of TMEM175 in lysosomes by utilizing whole‐endolysosome patch‐clamp recordings [35]. Notably, DCPIB, a synthetic agonist of TMEM175 [15], did not elicit any currents in TMEM175 knockout (KO) COS1 cells overexpressed with EGFP‐L156P (Figure 1J–L). Since the expression pattern of EGFP‐L156P resembled the ER network, we co‐transfected EGFP‐L156P with a biomarker for the ER, BFP‐Sec61b, in COS1 cells. Indeed, EGFP‐L156P was substantially retained in ER, unlike EGFP‐TMEM175 (Figure 1M, N), which resulted in negligible currents on the lysosomal and plasma membrane of cells over‐expressing EGFP‐L156P (Figure 1J–L; and Figure S2A–D). Together, L156P is identified as a PD‐associated variant of TMEM175, which is depleted from lysosomes.

The lysosome targeting motif that often contains a dileucine (Leu‐Leu) sequence at the N‐ or C‐ terminus plays a critical role in the intracellular trafficking of lysosomal membrane proteins [2, 3, 8]. However, L156P is one of the residues in the fourth transmembrane segment of the first repeat (TM4‐1) (Figure 1A), far away from the N‐ and C‐ terminus. It is reasonable to speculate that the aberrant expression pattern of L156P could not be deciphered by the classic lysosome targeting motif. To understand the underlying mechanism, we generated all possible mutations of L156 by replacing leucine with other amino acid residues and assessed the intracellular expression pattern of these mutants. Interestingly, the mutant EGFP‐L156D exhibited a mis‐localization similar to that of EGFP‐L156P (Figure 2A–C). In cells over‐expressed with L156D, TMEM175 currents were absent from both the lysosomal and the plasma membranes (Figure 2D; and Figure S2C, D). Additionally, EGFP‐L156E was localized to both the ER and lysosomes (Figure 2E, F), suggesting it partially disrupts the expression pattern of TMEM175. The remaining mutants of L156 predominantly localized to lysosomes, even when positively charged residues were present (Figure 2G–J; and Figure S3A–M). Thus, in addition to L156P, substituting L156 with acidic amino acid also results in impaired lysosome expression of TMEM175.

**FIGURE 2:**
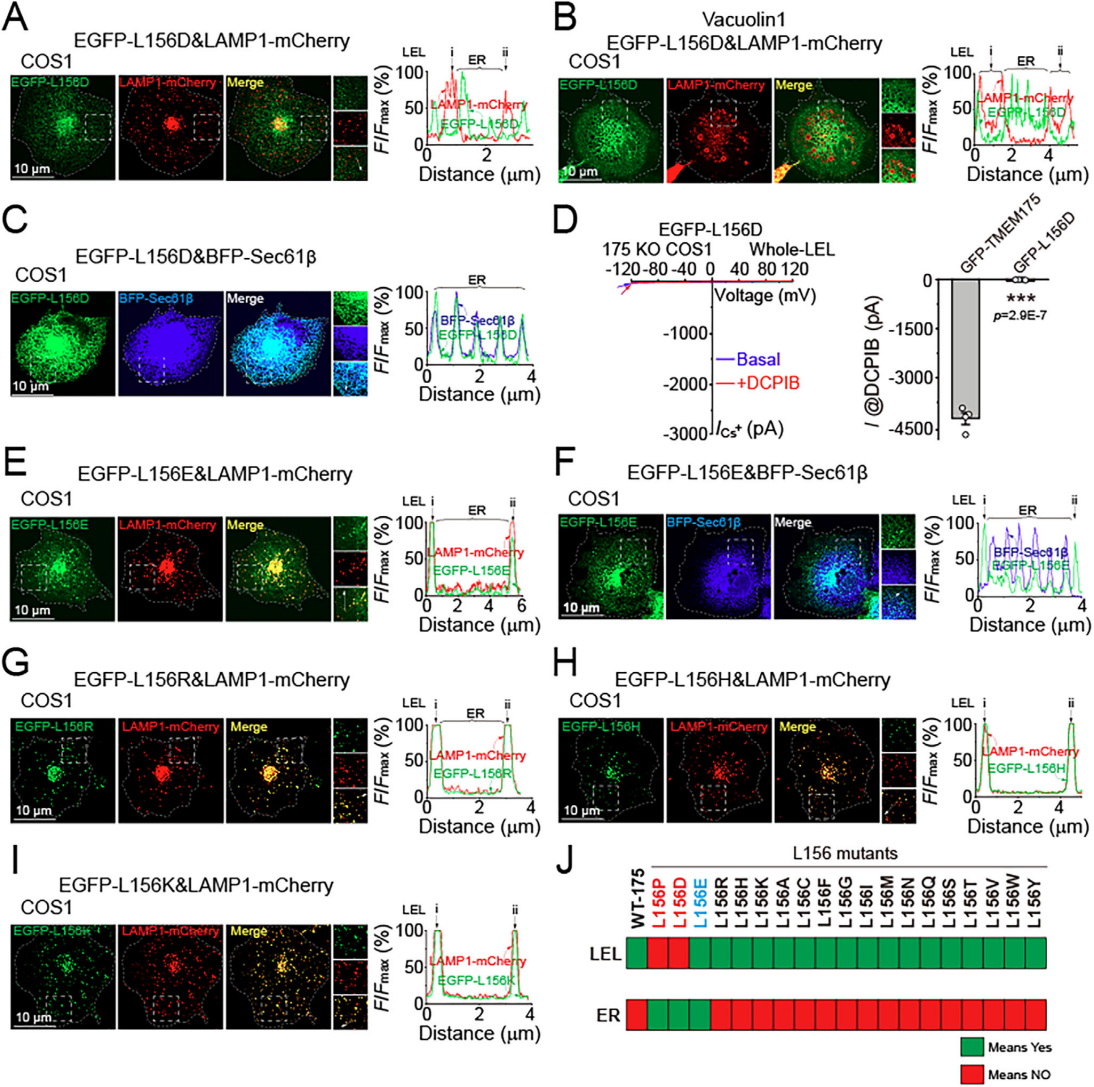
Disruption of lysosomal expression by EGFP‐L156D and EGFP‐L156E. (A and B) Representative images show the subcellular localization of EGFP‐L156D in TMEM175 KO COS1 cells in the absence (A) and presence of vacuolin‐1 (B). The graph to the Right of each group of images is a line scan through two separated lysosomes (LEL‐i and LEL‐ii) shown in the boxed region, indicating the intensity of EGFP‐L156D (green lines) and LAMP1‐mCherry (red lines) along the white arrow. Cells were treated with vacuolin‐1 for 12–18 h after transfection. Scale bar = 10 µm. n = 4. (C) Representative images show the co‐localization of EGFP‐L156D with BFP‐Sec61b in TMEM175 KO COS1 cells. ER are labeled in blue (BFP‐Sec61b). The graph to the Right of each panel shows the fluorescence intensity of a line scan (white arrows on the blown‐up image) through the double‐labeled object. Scale bar = 10 µm. n = 4. (D) Negligible Cs+ currents were potentiated by DCPIB from lysosomes in TMEM175 KO cells transfected with EGFP‐L156D. Data are mean ± s.e.m. from four independent experiments (n = 4). One‐way ANOVA. (E, F) Representative images show the co‐localization of EGFP‐L156E with LAMP1‐mCherry (E) and BFP‐Sec61b (F) in TMEM175 KO COS1 cells. Lysosomes are labeled in red (LAMP1‐mCherry), and ER are labeled in blue (BFP‐Sec61b). The graph to the Right of each panel shows the fluorescence intensity of a line scan (white arrows on the blown‐up image) through the double‐labeled object. Scale bar = 10 µm. n = 4. (G–I) Representative images show the co‐localization of EGFP‐L156R (G), EGFP‐L156H (H), and EGFP‐L156K (I) with LAMP1‐mCherry in TMEM175 KO COS1 cells. Scale bar = 10 µm. n = 4. (J) Summary of subcellular expression of L156 mutants.

### 2.3 The Cytosolic Part of TM4‐1 Determines the Lysosomal Localization of TMEM175

We next investigated the effects of the proline substitution at residues from V153 to G159 in TM4‐1 on the subcellular expression pattern of TMEM175. According to the resolved structure of the TMEM175 channel (PDB: 6WC9) [20], the segment of V^153^QALIVG^159^ is part of a cytosolic region in TM4‐1 that is entirely embedded in the lysosomal membranes (Figure 3A, B). Both V153 and G159 are oriented similarly to L156P on the alpha helix (Figure 3B). Both substitutions, V153P and G159P dramatically altered the intracellular expression pattern of EGFP‐TMEM175 (Figure 3C–F). While some colocalization with lysosomes was observed (Figure 3C–D), EGFP‐V153P substantially formed intracellular networks (Figure 3C), whereas EGFP‐G159P completely formed networks without any colocalization with lysosomes (Figure 3E, F). In a manner similar to EGFP‐L156P, both EGFP‐V153P and EGFP‐G159P also localized to the ER (Figure 3G, H). Additionally, Q154P, A155P, I157P, and V158P disrupted the lysosomal expression of EGFP‐TMEM175 and mis‐localized to the ER (Figure S4A–H). Thus, the cytosolic segment of TM4‐1 is responsible for determining the lysosomal expression of TMEM175.

**FIGURE 3:**
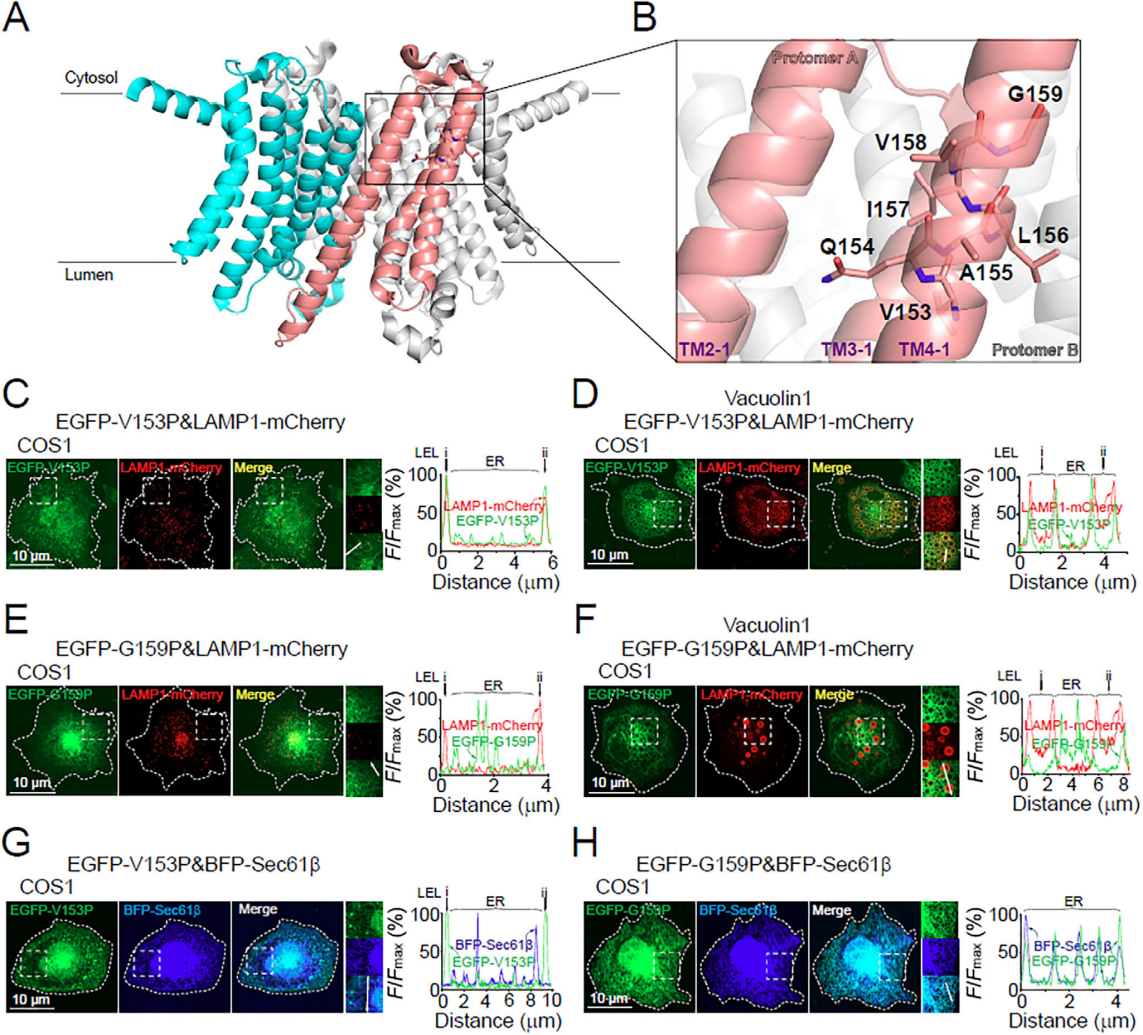
The crucial role of TM4‐1 in determining the lysosomal localization of TMEM175. (A, B) The illustration of the general (A) and detailed (B) structure of TM4‐1 in the repeat I of the TMEM175 channel (PDB:6WC9). Protomers A and B are shown in deep salmon plus cyan and grey, respectively. The TM1‐1 to TM6‐1 in repeat I are labeled in deep salmon, and the TM1‐2 to TM6‐2 in repeat II are labeled in cyan. The residues from V153 to G159 are exhibited as sticks. (C, D) Representative images show the subcellular localization of EGFP‐V153P in TMEM175 KO COS1 cells in the absence (C) and presence of vacuolin‐1 (D). The graph to the Right of each group of images is a line scan through two separated lysosomes (LEL‐i and LEL‐ii) shown in the boxed region, indicating the intensity of EGFP‐V153P (green lines) and LAMP1‐mCherry (red lines) along the white arrow. Cells were treated with vacuolin‐1 for 12–18 h after transfection. Scale bar = 10 µm. n = 4. (E and F) Representative images show the subcellular localization of EGFP‐G159P in TMEM175 KO COS1 cells in the absence (E) and presence of vacuolin‐1 (F). Scale bar = 10 µm. n = 4. (G and H) Representative images show the co‐localization of EGFP‐V153P (G) and EGFP‐G159P (H) with BFP‐Sec61b in TMEM175 KO COS1 cells. Scale bar = 10 µm. n = 4.

In the first repeat, TM3‐1 is the closest segment to TM4‐1 within the structure of the TMEM175 channel (PDB: 6WC9) [20, 22]. Notably, the residue of L156 within TM4‐1 and L112 within TM3‐1 directly face each other (Figure 4A, B). To explore the role of L112 in determining the lysosomal expression pattern of TMEM175, we created an EGFP‐L112P construct and evaluated its subcellular localization. Intriguingly, EGFP‐L112P was excluded from lysosomes (Figure 4C, D) and mis‐located to the ER (Figure 4E), similar to the behavior of EGFP‐L156P. Furthermore, negligible currents can be induced by TMEM175 agonist, DCPIB, from the lysosomal membrane and cell surface in TMEM175 KO cells over‐expressed with EGFP‐L112P (Figure 4F and Figure S5A), indicating that this mutant was trapped in the ER. In contrast, EGFP‐L112A remained on the lysosomal membrane and showed no ER localization (Figure S5B–D), which was reminiscent of the expression pattern of EGFP‐L156A (Figure S3A), suggesting that disrupting the interaction between L156:L112 by replacing with alanine doesn`t impair the lysosomal expression of EGFP‐TMEM175. Additionally, we assessed the effects of the proline substation of A115 (corresponding to V153) and L109 (corresponding to G159) in TM3‐1 (Figure 4B) on the subcellular expression of EGFP‐TMEM175. EGFP‐A115P still resided on the lysosomal membranes without any expression in the ER (Figure 4G–I), while EGFP‐L109P was absent from the lysosome and aberrantly targeted in the ER (Figure 4J–L). Therefore, TM3‐1 is also involved in determining the lysosomal expression of EGFP‐TMEM175.

**FIGURE 4:**
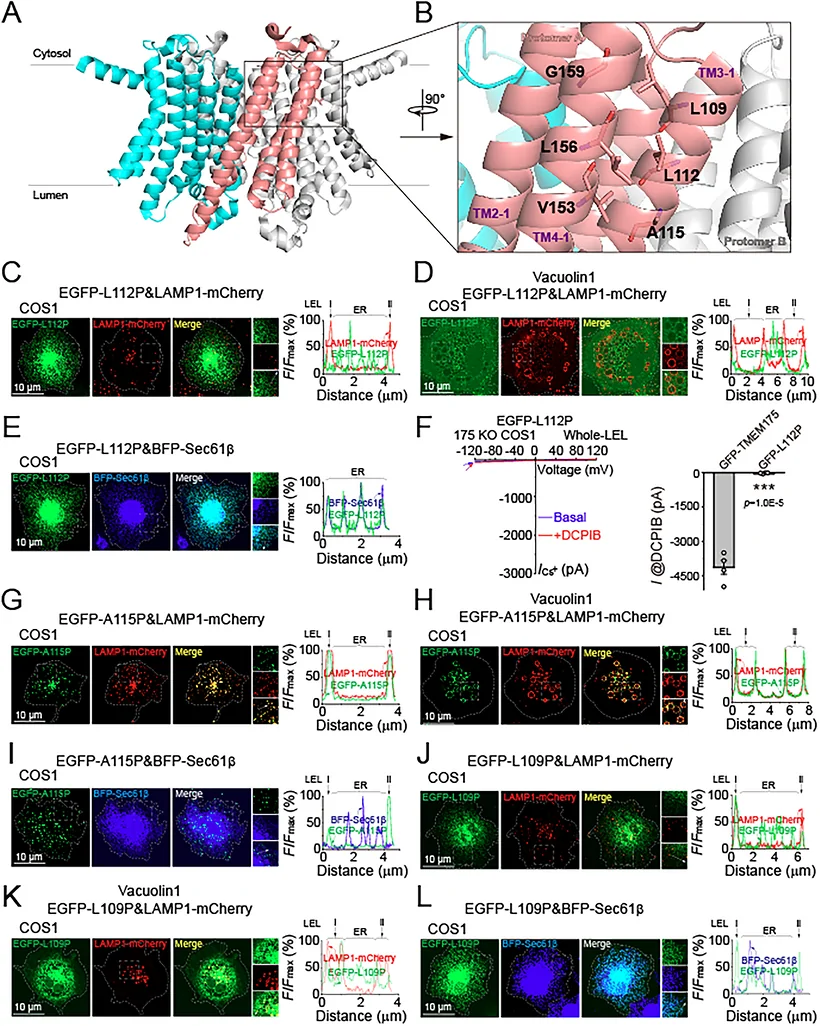
The involvement of TM3‐1 in determining the subcellular expression of TMEM175. (A, B) The illustration of the general (A) and detailed (B) structure of the interface between TM3‐1 and TM4‐1 in the repeat I of the TMEM175 channel (PDB:6WC9). Protomers A and B are shown in deep salmon plus cyan and grey, respectively. The TM1‐1 to TM6‐1 in repeat I are labeled in deep salmon, and the TM1‐2 to TM6‐2 in repeat II are labeled in cyan. The residues are exhibited as sticks. (C, D) Representative images show the subcellular localization of EGFP‐L112P in TMEM175 KO COS1 cells in the absence (C) and presence of vacuolin‐1 (D). The graph to the Right of each group of images is a line scan through two separated lysosomes (LEL‐i and LEL‐ii) shown in the boxed region, indicating the intensity of EGFP‐L112P (green lines) and LAMP1‐mCherry (red lines) along the white arrow. Cells were treated with vacuolin‐1 for 12–18 h after transfection. Scale bar = 10 µm. n = 4. (E) Representative images show the co‐localization of EGFP‐L112P with BFP‐Sec61b in TMEM175 KO COS1 cells. Scale bar = 10 µm. n = 4. (F) Negligible currents were potentiated by DCPIB from lysosomes in TMEM175 KO cells transfected with EGFP‐L112P. Data are mean ± s.e.m. from four independent experiments (n = 4). One‐way ANOVA. (G, H) Representative images show the lysosomal localization of EGFP‐A115P in TMEM175 KO COS1 cells in the absence (G) and the presence of vacuolin‐1 (H). Scale bar = 10 µm. n = 4. (I) Representative images show the subcellular localization of EGFP‐A115P with BFP‐Sec61b in TMEM175 KO COS1 cells. n = 4. (J, K) Representative images show the subcellular localization of EGFP‐L109P in TMEM175 KO COS1 cells in the absence (J) and presence of vacuolin‐1 (K). Scale bar = 10 µm. n = 4. (L) Representative images show the co‐localization of EGFP‐L109P with BFP‐Sec61b in TMEM175 KO COS1 cells. Scale bar = 10 µm. n = 4.

### 2.4 TM4‐2 in the Second Repeat is Essential for the Expression of TMEM175 in Lysosomes

Human TMEM175 possesses a two‐repeat structure, with each repeat consisting of six transmembrane segments (TM1‐TM6). Given the significant sequence similarity between two repeats [20, 22], we identified the corresponding residue in TM4‐2 as L391 to the residue of L156 in TM4‐1. Similarly, the corresponding residue in TM5‐2 was found to be L423, related to L112 in TM3‐1 (Figure S6A). To investigate the critical role of TM4‐2 in the lysosomal expression of TMEM175, we evaluated the subcellular pattern of L391P, L391D, and L423P. Analogous to EGFP‐L156P and EGFP‐L156D, both EGFP‐L391P and EGFP‐L391D also impaired the lysosomal expression of TMEM175 (Figure S6B–G). EGFP‐L391P was found in both the ER and lysosomes (Figure S6B–D), while EGFP‐L391D completely deprived the lysosome localization of TMEM175 (Figure S6E–G). DCPIB‐elicited currents were prominent in lysosomal membranes and on the cell surface in cells over‐expressing EGFP‐L391P (Figure S6H–M), although the current amplitude was much smaller than the wild‐type TMEM175 (Figure S6J–M). Contrarily, no currents can be activated by DCPIB in cells transfected with EGFP‐L391D (Figure S6I, J, L, M). Consistent with EGFP‐L112P, the mutant of EGFP‐L423P also altered the lysosomal expression of TMEM175 and was detected in both ER and lysosomes (Figure S7A–C). Thus, TM4‐2 is indispensable for the lysosomal expression of TMEM175.

### 2.5 The Lysosomal Localization of L156P is Promoted by Wild‐Type TMEM175

To investigate the subcellular expression pattern of L156P in heterozygous PD patients, we co‐expressed EGFP‐L156P with wild‐type TMEM175‐mCherry (WT‐mCherry) in TMEM175 KO COS1 cells. We labeled the lysosomes using immunostaining for LAMP1. We observed that the co‐transfection of WT‐mCherry significantly restored the lysosomal expression of EGFP‐L156P compared to EGFP‐L156P alone (Figure 5A, B). Specifically, equivalent co‐transfection of WT‐mCherry (EGFP‐L156P:WT‐mCherry = 1:1) significantly restored the lysosomal expression of EGFP‐L156P (7.29%±1.41%, n = 6 for EGFP‐L156P vs 50.64%±2.78%, n = 6 for EGFP‐L156P&WT‐mCherry; Figure 5A, B; and Figure S8A). This suggests that L156P has multiple subpopulations in heterozygous patients, as WT‐mCherry did not completely restore the lysosomal expression of EGFP‐L156P.

**FIGURE 5:**
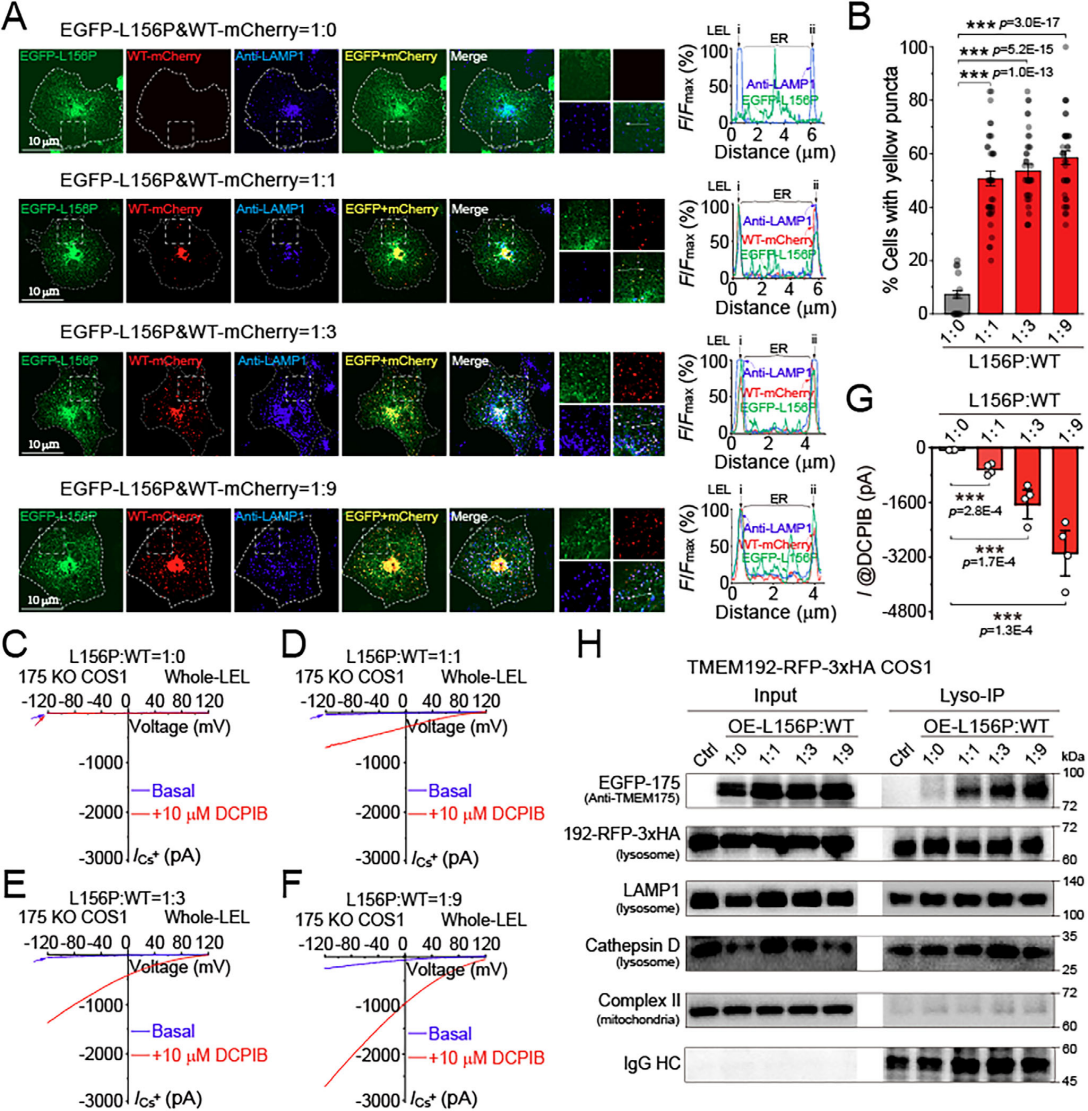
The promotion in the lysosomal expression of EGFP‐L156P by co‐expressing TMEM175‐mCherry. (A) Representative images show the subcellular localization of EGFP‐L156P in TMEM175 KO COS1 cells co‐transfected with WT TMEM175‐mCherry in various ratios. The graph to the Right of each group of images is a line scan through two separated lysosomes (LEL‐i and LEL‐ii) shown in the boxed region indicating the intensity of EGFP‐L156P (green lines), WT‐mCherry (red lines), and LAMP1 (blue lines) along the white arrow. Lysosomes were detected by immunostaining against LAMP1. Scale bar = 10 µm. (B) Quantification of percentage cells with the lysosomal expression of EGFP‐L156P in experiments shown in (A). Data are mean ± s.e.m. from four independent experiments (n = 4). 200–300 cells were selected for the analysis. One‐way ANOVA. (C–G) DCPIB‐elicited currents from lysosomes in TMEM175 KO cells co‐transfected with EGFP‐L156D and WT TMEM175‐mCherry in various ratios. Data are mean ± s.e.m. from six independent experiments (n = 6). One‐way ANOVA. (H) Western blot from lysosomal fractions of COS1 cells stably expressing TMEM192‐mRFP‐3xHA after co‐transfecting with EGFP‐L156P and WT TMEM175‐mCherry for 24 h. These fractions were immune‐isolated with magnetic beads coated with an anti‐HA antibody that recognizes the 3xHA epitope in TMEM192‐mRFP‐3xHA. n = 3.

We further assessed the impact of different transfection ratios on the lysosomal expression of EGFP‐L156P and found that a 75% ratio (EGFP‐L156P:WT‐mCherry = 1:3) and a 90% ratio (EGFP‐L156P:WT‐mCherry = 1:9) of WT‐mCherry increasingly promoted more EGFP‐L156P back to lysosomes (Figure 5A, B; and Figure S8A), This indicates that WT‐mCherry enhances the lysosomal expression of EGFP‐L156P, which can be attributed to the homo‐dimeric composition of the TMEM175 channel [20, 22]. Additionally, the DCPIB‐induced currents on the lysosomal membrane in TMEM175 KO cells co‐transfected with EGFP‐L156P and WT‐mCherry were restored with a gradual increase observed (Figure 5C–G). The Lyso‐IP results further corroborated that the quantity of TMEM175 in lysosomes was increased accordingly (Figure 5H). Notably, not only was the lysosomal pattern of EGFP‐L156P restored by WT‐mCherry, but EGFP‐L391P was also rescued by the co‐transfection (Figure S9A–C), although the enhancement for EGFP‐L391P was not as pronounced as for EGFP‐L156P.

### 2.6 Chemical Chaperones can Assist in the Folding and ER Exit of L156P

The most well‐known cystic fibrosis mutant, CFTR‐DF508, substantially accumulates in the ER [4, 36]. A variety of chemical chaperones [36, 37, 38] have been developed to prevent the degradation of CFTR‐DF508 and promote its exit from the ER [39]. After establishing that EGFP‐L156P is also aberrantly accumulated in the ER, we explored whether we could use chemical chaperones to help with the folding and facilitating the trafficking of EGFP‐L156P to lysosomes, potentially improving the disease condition. This approach is novel as it not only introduces the concept of precision medicine for treating PD but also repurposes established drugs for use in another disease besides cystic fibrosis. We evaluated two prominent CFTR correctors, Corrector C4 (Corr‐4a; Figure 6A) and VX‐809 (Lumacaftor; Figure S10A) [36, 38], and observed that a significantly greater portion of EGFP‐L156P was re‐located to the lysosome (Figure 6B, C; and Figure S10B, C). Additionally, the Lyso‐IP results exhibited that the lysosomal expression of EGFP‐L156P in the presence of Corr‐4a was significantly increased (Figure 6D). To test if the restored lysosome form of EGFP‐L156P was a functional channel, we conducted the whole‐endolysosomal patch‐clamp recording in TMEM175 KO cells overexpressed with EGFP‐L156P after treatment with the correctors. We found that Corr‐4a treatment significantly elevated the DCPIB‐induced currents on lysosomal membranes (I = 19.3±1.4, n = 6 for the control group vs I/I0 = 234.2±48.2, n = 6 for the Corr‐4a‐treatment group; Figure 6E, G). Furthermore, Corr‐4a dramatically restored the lysosomal acidification in EGFP‐L156P‐expressed TMEM175 KO cells as well (Figure 6H, I), suggesting that the physiological functionalities of the restored lysosome form of EGFP‐L156P are improved by the correctors. Overall, our results demonstrated that EGFP‐L156P could be rescued by repurposing some of the established chemical chaperones of CFTR‐*Δ*F508.

**FIGURE 6:**
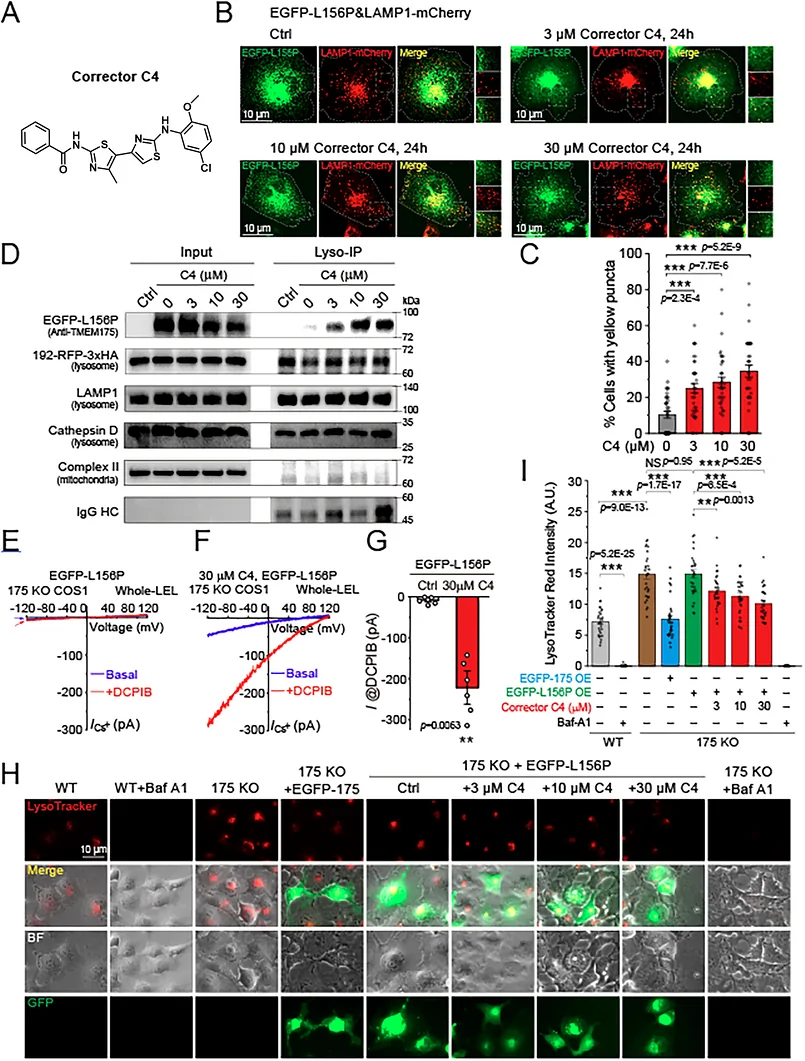
The assistance in the folding and ER exit of EGFP‐L156P by chemical chaperones. (A) Chemical structure of the Corrector C4 (Corr‐4a). (B) Representative images show the co‐localization of EGFP‐L156P with LAMP1‐mCherry in TMEM175 KO COS1 cells treated with the Corrector C4. Scale bar = 10 µm. (C) Quantification of percentage cells with the lysosomal expression of EGFP‐L156 in experiments shown in (B). Data are mean ± s.e.m. from six independent experiments (n = 6). 200–300 cells were selected for the analysis. One‐way ANOVA. (D) Western blot from lysosomal fractions of COS1 cells stably expressing TMEM192‐mRFP‐3xHA after with EGFP‐L156P for 24 h in the application of the Corrector C4. These fractions were immune‐isolated with magnetic beads coated with an anti‐HA antibody that recognizes the 3xHA epitope in TMEM192‐mRFP‐3xHA. n = 3. (E–G) DCPIB‐elicited currents from lysosomes in TMEM175 KO cells transfected with EGFP‐L156P after treatment with the Corrector C4. Data are mean ± s.e.m. from six independent experiments (n = 6). One‐way ANOVA. (H) Representative images of the LysoTrackerTM Red staining in TMEM175 KO COS1 cells overexpressed with EGFP‐L156P after treatment with the Corrector C4. Baf‐A1 was the positive control for modulating lysosome acidification. Scale bar = 10 µm. (I) Quantification of the LysoTrackerTM Red intensity in cells shown in (H). Data are mean ± s.e.m. from six independent experiments (n = 6). 400–600 cells were randomly selected for the analysis. One‐way ANOVA.

### 2.7 A Bifunctional Chemical Rescues the Aberrant Expression and Potentiates the Channel Function of EGFP‐L156P in Lysosomes

We recently employed the whole‐endolysosomal patch‐clamp recording method to identify small‐molecule agonists for lysosomal TMEM175 channels [15, 18]. We screened COS1 cells transiently expressing human TMEM175 channels with 200 compounds (Figure 7A) selected from the Library of Pharmacologically Active Compounds (LOPAC) [40], the same library of chemicals that were previously examined on TRPML1 and TPC2 channels [41], and 10 compounds randomly selected from our synthesized chemical library. Among the positive hits, we identified 20 compounds, including DCY‐09‐X (Figure 7B), significantly active EGFP‐TMEM175 in lysosomes with excellent specificity (Figure 7C–E). We also explored whether we could use a TMEM175 agonist to rescue the lysosomal expression of EGFP‐L156P. To our surprise, DCY‐09‐X treatment significantly restored the lysosomal expression (Figure 7F–H; and Figure S11A) and elevated the DCPIB‐induced currents on lysosomal membranes in TMEM175 KO cells overexpressed with EGFP‐L156P (Figure 7I–K), indicating that the TMEM175 agonist could rescue the aberrant expression and physiological function of L156P. Furthermore, DCY‐09‐X significantly restored the lysosomal acidification in EGFP‐L156P‐expressed TMEM175 KO cells as well (Figure 7L, M), suggesting that the physiological functionalities of the restored lysosome form of EGFP‐L156P are improved by the agonist. Therefore, DCY‐09‐X is a bifunctional molecule that not only activates the TMEM175 channels but also assists the mutant L156P in reaching the lysosomes and restoring the lysosomal acidification, which provides an intriguing and promising lead compound for further drug discovery of PD treatment.

**FIGURE 7:**
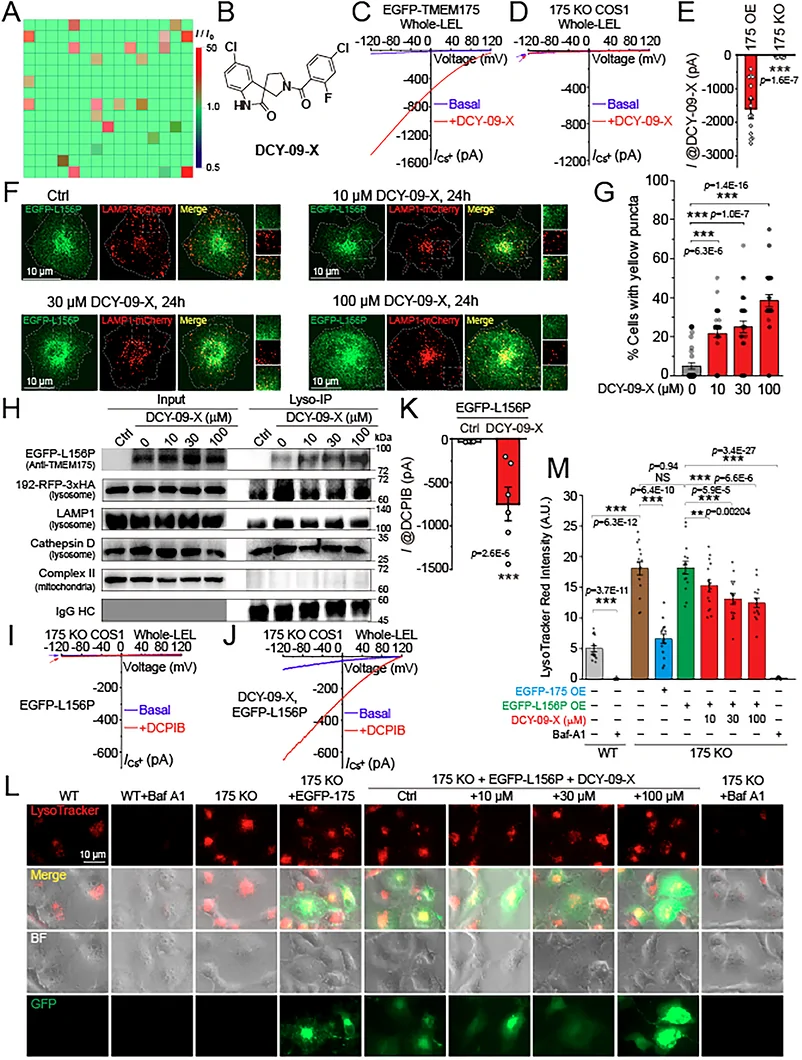
The rescue effects of TMEM175 agonist on EGFP‐L156P. (A) Screening of the compound library with whole‐endolysosomal patch‐clamp recordings in COS1 cells transiently expressed with EGFP‐TMEM175. Each square represented the average folds of the increased currents from lysosomes expressing EGFP‐TMEM175 in the application of individual compounds. The agonists are indicated in Red, and the compounds without any effects are shown in Green. (B) Chemical structure of the DCY‐09‐X. (C) Representative *I–V* plots of the lysosomal EGFP‐TMEM175 currents in the presence of DCY‐09‐X. (D) DCY‐09‐X induced negligible currents from the lysosome in TMEM175 KO COS1 cells. (E) The current amplitude in experiments shown in (C, D). Data are mean ± s.e.m. from at least six independent experiments (n≥6). Student's *t* test. (F) Representative images show the co‐localization of EGFP‐L156P with LAMP1‐mCherry in TMEM175 KO COS1 cells treated with the DCY‐09‐X. Scale bar = 10 µm. (G) Quantification of percentage cells with the lysosomal expression of EGFP‐L156 in experiments shown in (F). Data are mean ± s.e.m. from six independent experiments (n = 6). 200–300 cells were selected for the analysis. One‐way ANOVA. (H) Western blot from lysosomal fractions of COS1 cells stably expressing TMEM192‐mRFP‐3xHA after the transfection of EGFP‐L156P for 24 h in applying DCY‐09‐X. These fractions were immune‐isolated with magnetic beads coated with an anti‐HA antibody that recognizes the 3xHA epitope in TMEM192‐mRFP‐3xHA. n = 3. (I–K) DCPIB‐elicited currents from lysosomes in TMEM175 KO cells transfected with EGFP‐L156P after treatment with DCY‐09‐X. Data are mean ± s.e.m. from six independent experiments (n = 6). One‐way ANOVA. (L) Representative images of the LysoTrackerTM Red staining in TMEM175 KO COS1 cells overexpressed with EGFP‐L156P after treatment with DCY‐09‐X. Baf‐A1 was the positive control for modulating lysosome acidification. Scale bar = 10 µm. (M) Quantification of the LysoTrackerTM Red intensity in cells shown in (L). Data are mean ± s.e.m. from six independent experiments (n = 6). 400–600 cells were randomly selected for the analysis. One‐way ANOVA.

## 3 Discussion

The loss‐of‐function variants in human genes that encode ion channels and transporters are common and play a significant role in the pathogenesis of various diseases. However, trafficking‐deficient variants have not yet been studied as extensively. While the truncated and frame‐shifted variants usually cause the trafficking deficiency, the missense variants in several ion channels and transporters have also been found to be retained in the ER. In our research on lysosomal ion channel, we identified the first trafficking‐deficient variant in TMEM175, which encodes a proton and potassium channel in lysosomes. This variant is a missense mutation that substitutes the proline for leucine at position 156 of the TM4‐1 segment in TMEM175. The cytosolic part of both TM4‐1 and TM4‐2 are pivotal for determining the lysosomal expression of the TMEM175 channel. Genetically, L156P can be partially rescued by co‐transfection of wild‐type TMEM175, and pharmacologically, it was substantially restored by the application of chemical chaperones and synthetic agonists of TMEM175 as well. Our work emphasizes the importance of understanding the potential disease mechanism for individual variants in TMEM175 to encourage precision medicine. The identified compounds herein would pave the way for developing promising therapeutic strategies for clinical variants in TMEM175 with unique pathogenesis.

The sorting of ion channel proteins to lysosomes is facilitated by special signals present within the N‐ and C‐termini of the proteins [3, 8, 42]. One example of a lysosome‐targeting motif is the dileucine‐based signal, which fits [DE]XXXL[LI] or DXXLL consensus sequences recognized by adaptor proteins that coat peripherally associated with the cytosolic face of membranes [3]. For instance, the dileucine‐motif‐containing TRPML1 channel in humans bears L^15^L^16^ and L^577^L^578^ in their cytosolic tails, which is responsible for the lysosomal localization of this channel [8, 43, 44]. Although a dileucine motif of L^499^L^500^ does exist at the C‐terminus of TMEM175, it is dispensable to determine the lysosomal expression of this channel (data not shown). The L156 variant in TMEM175 is reminiscent of a mutant of both dileucine motifs (L^15^L/AA&L^577^L/AA) in TRPML1, which leads to a loss of lysosomal expression [43, 44]. However, the topology of TMEM175 suggests that L156 is unlikely to be involved in the lysosome targeting motif, as L156 resides in the transmembrane helix of TM4‐1, which may not be accessible to the adaptor proteins. Most mutants of L156, except for L156P/D/E, remain localized in the lysosome, excluding this explanation for TMEM175.

Proline, as a unique amino acid, would create a flexible kink or hinge within the transmembrane helix. Typically, a substitution of proline in the TM domain usually results in changes to protein folding or conformation, ultimately altering the properties of various ion channels [45, 46, 47, 48, 49]. Since L156 resides in TM4‐1, protein folding or confirmation issues may exist in the mutational channel of L156P. A proline scan in TMEM175 reveals that the cytosolic segment of TM4‐1 and TM3‐1 is responsible for the lysosomal localization. Additionally, within the repeat II, L391 and TM4‐2 are crucial for the normal expression of TMEM175. In contrast to the lysosomal depletion observed in L156P, the mutant of L391P and L423P exhibited multiple subpopulations in both lysosomes and ER, which could be interpreted with the similarity rather than identity between two repeats in TMEM175.

In heterozygous PD patients carrying the L156P mutation (TMEM175+/L156P), the dimeric channels formed by TMEM175, in principle, are composed in three scenarios at least: WT:WT, L156P:L156P, and WT:L156P. In the heterogenous expression system, we observed that wild‐type TMEM175 can help the amount of L156P reach the lysosomal membrane (Figure 4A). Since WT and L156P have the chance to pair up when they randomly assemble into dimeric channels, a WT subunit may act as a scaffold that promote the lysosome expression of the L156P subunit by forming the heterozygous WT:L156P channel. However, theoretically, the homozygous L156P:156P channel in patients is accumulated in the ER, despite the presence of a WT allele exists in the genome. In this homozygous scenario, the small molecular chaperone can be beneficial. Pharmacological chaperones hold promise as a therapeutic approach for certain diseases by interacting with mutant proteins to correct their folding and trafficking defects [37]. For example, in cystic fibrosis, corrector C4 and VX‐809 directly bind to the different domains of CFTR‐*Δ*F508 and thermodynamically stabilize the protein [36, 38]. These correctors can assist the cystinosin(7*Δ*) mutant in the proper folding and trafficking as well [9]. It is speculated that pharmacological correctors could function by directly stimulating either the folding of L156P or its ER exit. Our work indicates that small molecular correctors can be further repurposed [50] to treat PD, in addition to cystic fibrosis and cystinosis.

Strikingly, we identified a novel chemical molecule, DCY‐09‐X, that not only promotes the lysosome expression of L156P but also activates the TMEM175 channels. This bifunctional agonist of TMEM175 could first bring the ER‐retained L156P back to the lysosome and then it robustly potentiates the function of the mutant channel that is restored on lysosomal membranes. The bifunctional chemical molecule may offer a more advanced treatment for PD carrying L156P compared to existing correctors. Recently, bifunctional molecules, such as proteolysis targeting chimera (PROTAC) degraders and molecular glue chemicals, are hot spots in emerging therapies for drug development [51, 52]. The bifunctionality of both PROTAC degraders and molecular glue chemicals originates from the simultaneous binding to two target proteins. Nevertheless, our “chaperone plus agonist” bifunctional compounds perhaps target only one protein by binding to TMEM175. Further studies are needed to explore the structure basis and molecular mechanism underlying our bifunctional compounds.

In this study, we demonstrate that the L156P variant of human TMEM175 represents a bona fide trafficking‐deficient mutation. Rather than impairing channel activity per se, L156P is mis‐localized to the ER, leading to a loss of functional expression on lysosomal membranes. These findings establish that defective intracellular trafficking, independent of intrinsic channel dysfunction, can serve as a pathogenic mechanism for TMEM175‐associated PD. By extending the concept of trafficking deficiency from plasma membrane channelopathies to lysosomal ion channels, our work provides a conceptual framework for understanding the mechanistic diversity of TMEM175 variants. This distinction has important implications, as it suggests that PD‐associated mutations in TMEM175 may not constitute a uniform class of loss‐of‐function variants [14, 26, 33], but instead encompass mechanistically distinct subtypes requiring tailored therapeutic strategies. Importantly, our identification of chemical chaperones and a bifunctional TMEM175 agonist that restore both trafficking and function of L156P highlights intracellular trafficking as a druggable process. These findings reinforce the idea that correcting protein localization, rather than solely modulating channel activity, may represent an effective therapeutic avenue for specific genetic forms of PD.

Despite these findings, our study still has several limitations. First, our study primarily relies on heterologous expression systems, which may not fully recapitulate the complex cellular environment of neurons or glial cells in PD. Second, although we demonstrate restoration of lysosomal localization and function in vitro, the in vivo efficacy and pharmacokinetics of the identified compounds remain to be determined. In addition, while our data support a trafficking‐deficient mechanism for L156P, it is possible that other TMEM175 variants may involve mixed mechanisms, including both trafficking defects and intrinsic functional impairment. A systematic classification of TMEM175 variants will therefore be necessary to fully understand genotype–phenotype relationships.

## 4 Methods

### 4.1 Molecular Biology

The pEZ‐M29‐EGFP‐hTMEM175 construct (GeneCopoeia EX‐V0772‐M29) was purchased from GeneCopoeia Company, which has been described in our previous work [18]. TMEM175 mutants were generated with QuikChange lightning II site‐directed mutagenesis kit (Agilent) and confirmed by DNA sequencing.

### 4.2 Mammalian Cell Lines

COS1 cells (WT and TMEM175 KO, Cyagen Biosciences Company, ATCC, CRL‐1650) has been described in our previous work [18]. Briefly, cells were cultured in Dulbecco's Modified Eagle Medium (DMEM, Thermo Fisher Scientific) with 10% FBS (Gibco). They were transfected with Lipofectamine^TM^ 3000 (Invitrogen) kit. All cells were used at low passages without further authentication or testing for mycoplasma contamination.

### 4.3 Generation of TMEM175 KO Cell Line with CRISPR‐Cas9

TMEM175 KO COS1 cells were generated by using a CRISPR/Cas9 system and purchased from the Cyagen Biosciences Company, which has been validated and described in our previous work [18]. After receiving TMEM175 KO cells, we confirmed the intended genetic disruption with sequencing, western blotting, and the whole‐endolysosome recording [18]. And the primers used for sequencing are: TMEM175‐F: ACACATGATCTGCAAAGACCCT, TMEM175‐R: TCCGATTCTGC GGAGCTTTC. The expected amplicon size generated by TMEM175‐F and TMEM175‐R was 4250 bp at an annealing temperature of 62.0°C. The GenBank accession number for TMEM175 is NC_132930.1. Primer‐BLAST analysis (https://www.ncbi.nlm.nih.gov/tools/primer‐blast/index.cgi?LINK_LOC=BlastHome) showed that TMEM175‐F and TMEM175‐R specifically bind to NC_132930.1. Consistently, agarose gel electrophoresis of the PCR products from WT and TMEM175 KO cells demonstrate single bands of 4250 and 1146 bp, respectively. Together, these results indicate that our primers specifically amplify the intended targets without detectable non‐specific amplification.

### 4.4 Generation of TMEM192‐mRFP‐3xHA COS1 Stable Cell Lines

HEK293T cells were seeded into 10 cm dishes and cultured overnight. The transfection was performed with Lipofectamine 3000 according to the manufacturer's instructions at a ratio of 4:2:3 for the pLJM1‐TMEM192‐mRFP‐3xHA (addgene plasmid #134631), VSVG, and PAX2. After 48 h of transfection, the supernatant containing the virus was collected using a 5 mL syringe and filtered through a 0.45 µm filter. To generate a stable cell line, low‐passage COS1 cells were seeded into 3.5 cm dishes at a density of 70%–80% and infected with approximately 1/3 of the virus volume for 48 h. Then, 1 µg/mL puromycin was added for screening for 4 days. To obtain a monoclonal cell line, each single clone was selected by serial dilution and gradually expanded.

### 4.5 Transient Transfection

All transfections were performed in COS1 or HEK293T cells that were approximately 80% confluent. This study used two transfection reagents, namely Lipofectamine LTX (Invitrogen, 15338100) and Lipofectamine 3000 (Invitrogen, L3000015) for different cells. The LTX reagent was used primarily for the transfection of constructs into COS1. In each transfection experiment, the mixture of plasmid DNA (4 µg) and Lipofectamine LTX was added to the cells in accordance with the manufacturer's instructions. After transfection for 24 h, the cells were digested with Trypsin and then seeded in culture dishes or plates for imaging, western blotting, or electrophysiology experiments 24–48 h after transfection. Lipofectamine 3000 was used for the transfection of HEK293T cells. According to the manufacturer's instructions and the size of the cell culture dish, the plasmid DNA was transferred into the cells to generate viruses used in stable cell lines.

### 4.6 Confocal Microscopy and Image Processing

All fluorescence imaging was performed using laser confocal acquisition (FV3000, Olympus). For live cell imaging, cells were seeded in 24‐well cell imaging culture plates and cultured for 24 h. Then FV3000 imaging could be adopted. For fixed cells, cells were seeded on glass coverslips coated with Poly‐L‐Lysine. After transfection and drug administration, the cells were washed three times with 1x PBS and fixed with 4% PFA. Then coverslips were mounted with Fluoromount‐G (Southern Biotech, cat. 0100–01) and collected for imaging with an inverted confocal microscope (FV3000, Olympus) equipped with an oil 100 x objective lens. The filter sets FITC (excitation, 488 nm), RFP (excitation, 561 nm), and DAPI (excitation, 405 nm), were used for EGFP, mCherry, and BFP, respectively. During the application of laser confocal microscopy, it is necessary to adjust the appropriate fluorescence intensity and laser power and select a resolution of 2048 × 2048.

The co‐localization analysis is performed with ImageJ software (National Institutes of Health), which quantitatively calculates the fluorescence intensity based on the gray value in images. Drag the image into ImageJ and color split. Select two lysosomes as representatives, and calculate the fluorescence intensity. Copy the fluorescence intensity data and paste it to Origin 2018 (OriginLab, Northampton, MA). From there, calculate the fluorescence intensity percentage and create a curve graph that illustrates the relationship between distance and fluorescence intensity percentage.

### 4.7 Immunofluorescence

Cells seeded on glass coverslips were washed with 1x PBS and fixed with 4% (v/v) paraformaldehyde at room temperature for 15 min or 100% anhydrous methanol for 8 min at −20°C. Afterward, 0.3% Triton‐X 100 in block solution (PBS supplemented with 2% bovine serum albumin) was used for 1 h to permeate the cell membrane in the PFA fixation method. Cells were incubated with a primary antibody overnight at 4°C and then incubated with a secondary antibody at room temperature for 1 h. The primary antibody (anti‐human LAMP1 (1:200, DSHB, cat. H4A3)) and the secondary antibody (Goat anti‐Rabbit conjugated to Alexa FluorTM 405 (1:800, ThermoFisher, cat. A‐31556)) were diluted into the block solution proportionally as indicated. Coverslips were mounted on glass slides with Fluoromount‐G (Southern Biotech, cat. 0100–01). Images were acquired with an inverted confocal microscope (FV3000, Olympus) equipped with an oil 100 x objective lens and analyzed with ImageJ (NIH).

### 4.8 Lysosomal Immune‐Isolation and Immunoprecipitation (Lyso‐IP)

Based on the literature [9, 34], the general steps are: The day before the experiment, TMEM192‐mRFP‐3×HA COS1 stable cells transfected with EGFP‐L156P were seeded into 10 cm dishes. Cells were washed twice with pre‐chilled TBS (20 mm Tris, 150 mm NaCl, pH = 7.25) and then harvested using a cell scraper in 1 mL 1x TBS (containing protease (Roche, cat. 04693124001) and phosphatase inhibitors (Roche, cat. 10837091001)) on ice. Afterwards, cells were centrifuged at 1000 g, 4°C for 1 min. The pellet was resuspended with 1 mL pre‐chilled 1x TBS (containing protease and phosphatase inhibitors) and a 100 µL sample was split out as the input for western blotting. The remaining 900 µL was added with CaCl2 to a final concentration of 8 mm and homogenized in a Dounce homogenizer for approximately 25 strokes on ice. The supernatant was centrifuged at 1150 g, 4°C for 5 min. This step will obtain post‐nuclear supernatants dominantly containing lysosomes with a higher purity [34]. 35 µL anti‐HA magnetic beads (Thermo Scientific, cat. 88836) for each sample were pre‐equilibrated by washing with 1 mL pre‐chilled 1xTBS (containing protease, phosphatase inhibitors, and 8 mm CaCl_2_) on a shaker at 45 rpm, 4°C for 5 min. Afterward, the EP tube containing anti‐HA magnetic beads was placed on a magnetic stand to remove the 1x TBS solution. Repeat this pre‐equilibrated procedure twice. The post‐nuclear supernatants and pre‐equilibrated anti‐HA magnetic beads were incubated at 4°C for 30 min. The IP samples were washed on a magnetic stand three times with IP buffer 1x TBS (containing protease, phosphatase inhibitors, and 8 mm CaCl_2_). 100 µL of LDS sample buffer (Invitrogen, cat. NP0007) was added to the harvested IP samples for protein extraction. The IP products were used and analyzed by western blotting.

The protein extraction process for the input group is as follows. Input samples were centrifuged at 12 000 rpm, 4°C for 30 min, and the supernatant was removed. Then, add 90 µL of RIPA to the precipitate and lyse them on ice for 30 min, followed by centrifugation at 12 000 rpm, 4°C for 30 min. The supernatant was then collected, and 20 µL of 5×SDS loading buffer (YEASEN, cat. 20315ES05) was added and heated at 95°C in a water bath for 10 min, after which it was stored at −20°C for future use. The protein samples were separated by 10% SDS‐PAGE gel and transferred onto PVDF membranes for analysis. The membranes were incubated with 5% skimmed milk for 1 h at room temperature for blocking, and then incubated with primary antibodies at 4°C and secondary antibodies at room temperature respectively. An AffinityTM ECL kit (femtogram, cat. KF8003) was used to bind the membrane‐bound proteins, and the membrane was scanned using the automatic chemiluminescence imaging system (Tanon‐5200 Multimate).

The primary antibodies used in this study for western blotting are anti‐TMEM175 (1:1500, Proteintech, cat. 19925‐1‐AP), anti‐HA (1;1000, Thermo fisher, cat. 26183), anti‐Complex II (1:1000, Thermo fisher, cat. 459200), anti‐LAMP1 (Cell signaling Technology, cat. D2D11), anti‐Cathepsin D (1:1500, Abcam, cat. ab72915), anti‐Ig‐G (1:1000, Santa Cruz, cat. sc2025). The secondary antibodies used in this study for western blotting are goat anti‐mouse HRP (YEASEN, 1:10000, cat. 33201ES60) and goat anti‐rabbit HRP (YEASEN, 1:10000, cat. 33701ES60).

### 4.9 LysoTracker^TM^ Red Staining

To roughly estimate the acidity of lysosomes, cells were seeded in 24‐well plates and cultured overnight. After 8 h of incubation, the medium was replaced with a drug‐containing medium and treated for 24 h. Subsequently, 50 nm LysoTracker Red (Invitrogen, cat. L7528) was added to the complete medium and incubated at 37°C for 30 min. After incubation, the cells were washed twice with Tyrode's solution and maintained in Tyrode's solution for imaging. Images were captured using an inverted fluorescence microscope (Olympus IX73), and the fluorescence intensity of LysoTracker Red was quantified using ImageJ software (NIH).

### 4.10 Whole‐Endolysosome Patch‐Clamp Electrophysiology

We employed a modified patch‐clamp method to record endosomal and lysosomal channels [35]. Lysosomes were selectively enlarged with 1 m vacuolin‐1 (Millipore Sigma, cat. 673000) for 12–16 h. Enlarged endolysosomes were captured with a glass pipette. After reaching a giga‐seal, a break‐in step protocol with a few hundred mVs lasting 1–4 ms was applied to break the vacuolar membrane. The whole‐LEL configuration was verified by the re‐appearance of capacitance transients after a break‐in.

Unless otherwise stated, the pipette (luminal) solution for Cs^+^ current contained (in mM): 145 Cs^+^, 140 MSA^−^, 5 Cl^−^, 20 HEPES (pH adjusted to 7.20 with MSA); and the internal/cytosolic solution contained (in mM): 145 NMDG^+^, 140 MSA^−^, 5 Cl^−^, 20 HEPES (pH adjusted to 7.2 with MSA). All bath solutions were applied through a perfusion system, in which solutions were completely exchanged within a few seconds. Data were collected via an Axopatch 200B patch clamp amplifier, Digidata 1550B, and pClamp 10.2 software (Axon Instruments). Patch pipette electrodes had a resistance of 8–12 mΩ. Whole‐EL currents were digitized at 10 kHz and filtered at 2 kHz. Patches with a membrane resistance >3 GΩ were accepted. Lysosomal transmembrane potential (Vm) is defined as V_Cytosol_‐V_Lumen_ [8]. A ramp protocol (−120 to +120 mV, 200 ms, holding at 0 mV) was used to record the currents at 5s intervals. All experiments were conducted at room temperature (22C–25C) with 1 m KCl Agar Bridge connecting the recording chamber to a ground wire. All data were analyzed with pClamp 11.2 (Molecular Devices) and Origin 2018 (OriginLab, Northampton, MA).

### 4.11 Whole‐Cell Patch‐Clamp Electrophysiology

Whole‐cell recordings were performed in COS1 cells using pipette electrodes with 5–10 MΩ resistance. We make the glass electrode contact the cell membrane, release the positive pressure in the electrode, and gently apply the negative pressure to form a seal. Unless otherwise stated, the pipette (luminal) solution for Cs^+^ current contained (in mm): 145 Cs^+^, 140 MSA^−^, 5 Cl^−^, 20 HEPES (pH adjusted to 7.20 with MSA); and the internal/cytosolic solution contained (in mm): 145 NMDG^+^, 140 MSA^−^, 6 Cl^−^, 20 HEPES (pH adjusted to 7.2 with MSA). Whole‐cell currents were digitized at 10 kHz and filtered at 2 kHz. Patches with a membrane resistance > 1 GΩ were accepted. A ramp protocol (−120 to +120 mV, 200 ms, holding at 0 mV) was used to record the currents at 5s intervals. All experiments were conducted at room temperature (22C–25C) with a 1 M KCl Agar Bridge connecting the recording chamber to a ground wire. All data were analyzed with pClamp 11.2 (Molecular Devices) and Origin 2018 (OriginLab, Northampton, MA).

### 4.12 Synthesis of Key Compound DCY‐09‐X

The synthetic route of the target compounds is shown in Scheme 1. The commercially available 2‐(5‐chloro‐1*H*‐indol‐3‐yl)ethanamine hydrochloride intermediate **1a** was cyclized under the presence of paraformaldehyde and acetic acid to give the key intermediate **2a** through a classic Pictet‐Spengler reaction. The other key intermediate **4a** was synthesized from 4‐chloro‐2‐fluorobenzoic acid as the starting material. The condensation of intermediates **2a** and **4a** in the presence of triethylamine in THF produced the key intermediate **5a**, which was subsequently converted to the target compound DCY‐09‐X via an NBS‐mediated oxidative rearrangement. The detailed operations as follow:

**SCHEME 1:**
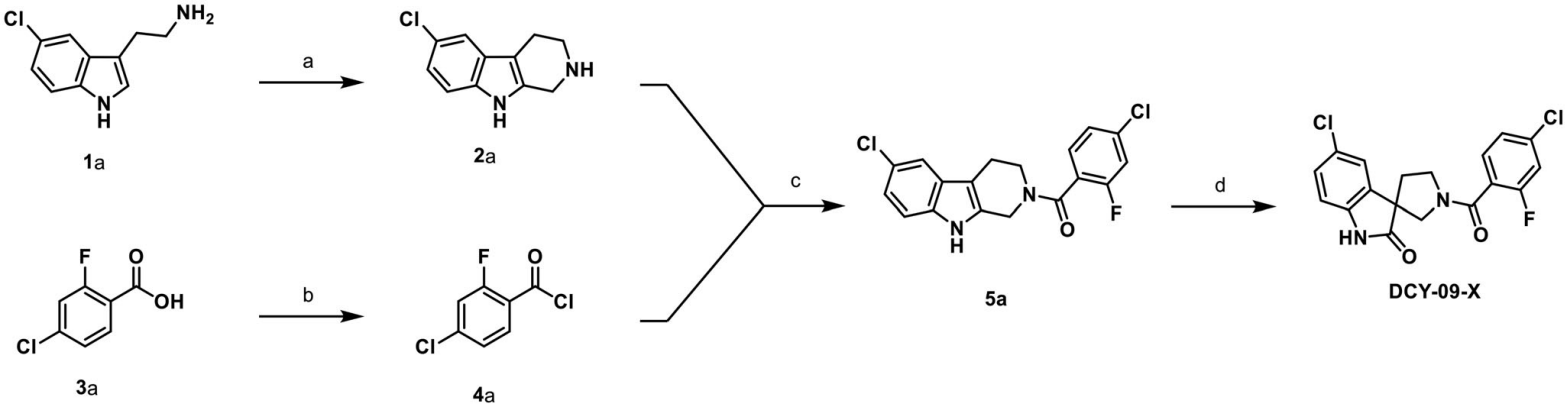
Synthesis of the key compound.^a^ Reaction conditions and reagents: (a) paraformaldehyde, glacial acetic acid, methanol, 80°C, 1 h; (b) oxalyl chloride, N, N‐dimethylformamide, dichloromethane, 0°C to room temperature, 2 h; (c) triethylamine, tetrahydrofuran, room temperature, 12 h; (d) N‐bromosuccinimide, tetrahydrofuran, acetic acid, water, 0°C.

2‐(5‐Chloro‐1*H*‐indol‐3‐yl) ethanamine hydrochloride **1a** (594 mg, 2.57 mmol) was dissolved in a solvent mixture (14 mL) of glacial acetic acid and methanol (2/5, v/v), and paraformaldehyde (93 mg, 3.08 mmol) was added. The reaction mixture was stirred for 2 h at 80°C and then cooled to room temperature. The mixture was basified to pH 9–10 using ammonium hydroxide solution and extracted with dichloromethane. The combined organic phase was washed with brine (15 mL), dried over Na_2_SO_4_, filtered and concentrated under reduced pressure. The crude product was triturated with diethyl ether and filtered to afford **2a** as a pale‐yellow solid (279 mg, 53% yield).

4‐Chloro‐2‐fluorobenzoic acid **3a** (200 mg, 1.15 mmol) was dissolved in 4 mL anhydrous dichloromethane, and cooled in an ice bath. Oxalyl chloride (485 µL, 5.73 mmol) was dissolved in dichloromethane (1 mL), and added dropwise to the above mixture under argon protection. After that, 1–2 drops of N, N‐dimethylformamide was added and then move it to room temperature to stir for 2 h. The solvents were removed under reduced pressure to obtain the colorless oil. The crude product 4a was used for the next step without further purification.

The intermediate **4a** (221 mg, 1.15 mmol) was dissolved in anhydrous tetrahydrofuran (5 mL), then intermediate **2a** (237 mg, 1.15 mmol) and triethylamine (630 µL, 4.58 mmol) were added sequentially. The reaction mixture was stirred at room temperature for 12 h. When the substrates disappeared, saturated solution of sodium bicarbonate (10 mL) was added to neutralize acid, then the resulting mixture was extracted with ethyl acetate (3 × 15 mL). The combined organic layer was washed with brine, dried over Na_2_SO_4_, filtered, and concentrated in vacuo. The residue was purified by column chromatography (DCM/MeOH = 100/1 to 25/1, v/v) to obtain key intermediate **5a** as a white solid (220 mg, 53% yield). ^1^H NMR (800 MHz, DMSO‐*d*6) δ 11.18 (s, 1H), 10.81 (s, 0.5H), 7.64 – 7.61 (m, 1.5H), 7.52 (t, *J* = 7.8 Hz, 1H), 7.50 (t, *J* = 7.8 Hz, 0.5H), 7.48 – 7.40 (m, 3H), 7.36 (d, *J* = 8.6 Hz, 1H), 7.30 (d, *J* = 8.5 Hz, 0.5H), 7.06 (dd, *J* = 8.6, 2.1 Hz, 1H), 7.04 (dd, *J* = 8.5, 2.1 Hz, 0.5H), 4.88 (s, 2H), 4.50 (s, 1H), 4.06 – 3.94 (m, 1H), 3.57 (t, *J* = 5.7 Hz, 2H), 2.79 (t, *J* = 5.9 Hz, 1H), 2.68 (t, *J* = 5.8 Hz, 2H). ^13^C NMR (201 MHz, DMSO‐*d*_6_) δ 164.1, 163.7, 157.8 (d, *J* = 249.2 Hz), 157.7 (d, *J*_F‐C_ = 249.5 Hz), 135.2 (d, *J*_F‐C_ = 10.3 Hz), 135.0 (d, *J*_F‐C_ = 10.2 Hz), 134.5, 134.4, 132.3, 132.2, 130.2 (d, *J*_F‐C_ = 4.6 Hz), 130.0 (d, *J*_F‐C_ = 4.5 Hz), 127.6, 125.4 (d, *J*_F‐C_ = 3.5 Hz), 125.4 (d, *J*_F‐C_ = 3.4 Hz), 123.3 (d, *J*_F‐C_ = 18.3 Hz), 123.3, 123.3 (d, *J*_F‐C_ = 19.0 Hz), 120.8, 120.8, 117.1, 117.0, 116.7 (d, *J*_F‐C_ = 25.2 Hz), 116.7 (d, *J*_F‐C_ = 25.3 Hz), 112.6, 112.6, 107.1, 106.5, 44.8, 44.6, 40.0, 40.0, 21.4, 20.4. HRMS (ESI) m/z: calcd for C_18_H_14_Cl_2_FN_2_O^+^ [M + H]^+^, 363.0462; found, 363.0463. Note: Due to the hindered rotation around the amide C─N bond, it leads to the formation of atropisomer and a pair of conformers. According to the ^1^H NMR spectrum, the ratio of the two conformations is about 2:1.

A solution of intermediate **5a** (200 mg, 0.55 mmol) in THF/AcOH/H_2_O mixed solvent (1/1/1, v/v/v, in total 18 mL) was treated with N‐bromosuccinimide (108 mg, 0.61 mmol) portionwise at 0°C over 20 min. The resulting mixture was stirred at 0°C for 100 min. The mixture was then quenched by the addition of saturated Na_2_CO_3_ (50 mL) and extracted with DCM. The combined organics were washed with saturated NaHCO_3_ and brine, dried over Na_2_SO_4_, filtered and concentrated to the crude residue, which was purified by column chromatography (DCM/MeOH = 100/2 to 20/1, v/v) to yield DCY‐09‐X (150 mg, 72%). ^1^H NMR (500 MHz, DMSO‐*d*_6_) δ 10.70 (s, 1H), 10.62 (s, 1H), 7.63 (dd, J = 9.7, 2.0 Hz, 1H), 7.57 (t, J = 7.8 Hz, 1H), 7.52 (dd, J = 9.7, 2.0 Hz, 1H), 7.48 (dd, J = 8.2, 7.4 Hz, 1H), 7.46 – 7.42 (m, 2H), 7.36 (dd, J = 8.3, 2.0 Hz, 1H), 7.31 – 7.28 (m, 2H), 7.25 (dd, J = 8.3, 2.2 Hz, 1H), 6.90 (d, J = 8.3 Hz, 1H), 6.84 (d, J = 8.2 Hz, 1H), 3.95 – 3.82 (m, 2H), 3.81 – 3.73 (m, 2H), 3.72 – 3.65 (m, 1H), 3.63 – 3.56 (m, 1H), 3.53 (s, 2H), 2.35 – 2.13 (m, 4H). ^13^C NMR (126 MHz, DMSO‐*d_6_*) δ 178.76, 178.12, 163.18, 163.03, 157.84 (d, J_F‐C_ = 249.9 Hz), 157.81 (d, J_F‐C_ = 250.3 Hz), 140.58, 135.19, 135.11, 133.69, 133.16, 130.08 (m, J_F‐C_ = 5.2 Hz), 128.23, 128.15, 125.91, 125.79, 125.28 (d, J_F‐C_ = 3.0 Hz), 125.22 (d, J_F‐C_ = 3.1 Hz), 123.95 (m, J_F‐C_ = 18.1 Hz), 123.20, 123.00, 116.80 (d, J_F‐C_ = 25.4 Hz), 116.77 (d, J_F‐C_ = 25.3 Hz), 111.02, 110.92, 55.11, 53.15, 52.44, 51.47, 46.55, 44.78, 35.49, 33.99. HRMS (ESI) m/z: calcd for C_18_H_14_Cl_2_FN_2_O_2_^+^ [M + H]^+^, 379.0411; found, 379.0412. Note: Due to the hindered rotation around the amide C─N bond, it leads to the formation of atropisomer and a pair of conformers. According to the ^1^H NMR spectrum, the ratio of the two conformations is about 1:1.

### 4.13 Reagents

DCPIB (Tocris Bioscience, cat. 1540); Corrector C4 (TargetMol, cat. T31014); VX‐809, Lumacafter (TargetMol, cat. T31014); vacuolin‐1 (Millipore Sigma, cat. 673000); CaCl2 (Sigma–Aldrich, cat. 102318667); DMSO (Sigma–Aldrich, cat. D2650); NaCl (Sigma–Aldrich, cat. 7647145); Bafilomycin‐A1 (MedChemExpress, cat. HY‐100558); Tris hydrochloride (Merck Millipore, cat. 648313); AffinityTM ECL kit (femtogram, cat. KF8003); skim milk (YEASEN, cat. 36120ES76); Primary Antibody Dilution Buffer (Beyotime, cat. P0256); 10x PBS (Solarbio, cat. P1022); LDS sample buffer (Invitrogen, cat. NP0007); anti‐HA magnetic beads (Thermo Scientific, cat. 88836); LysoTracker Red DND‐99 (Invitrogen, cat. L7528);4% PFA (BBI, cat. E672002); Lipofectamine LTX (Invitrogen, cat. 15338100); Lipofectamine 3000 (Invitrogen, cat. L3000015); Fluoromount‐G (SouthernBiotech, cat. 0100–01). anti‐LAMP1 (1:200, DSHB, cat. H4A3, for western blotting); anti‐TMEM175 (1:1500, Proteintech, cat. 19925‐1‐AP); anti‐HA (1:1000, Thermo fisher, cat. 26183); anti‐Complex II (1:1000, Thermo fisher, cat. 459200); anti‐LAMP1 (1:1000, Cell signaling Technology, cat. D2D11, for IF), anti‐Cathepsin D (1:1500, Abcam, cat. ab72915); anti‐Ig‐G (1:1000, Santa Cruz, cat. sc2025). goat anti‐rabbit (1:800, thermo Fisher, cat. A‐31556); goat anti‐mouse HRP (1:10000, YEASEN, cat. 33201ES60); goat anti‐rabbit HRP (1:10000, YEASEN, cat. 33701ES60).

### 4.14 Quantification and Statistical Analysis

All data were analyzed and plotted with Origin 2018 (OriginLab, Northampton, MA). For individual parametric samples, when assumptions of normality and equal variances were met, Student's unpaired *t*‐test were used; for normally distributed data with unequal variances, an unpaired two‐tailed Student's unpaired *t*‐test with Welch's correction is applied. For individual nonparametric samples, when the assumptions of normal distribution and homoscedasticity are fulfilled for correlated data, a paired Student's *t*‐test were used; in cases where these assumptions are not met, we resorted to the Wilcoxon matched‐pairs signed rank test. One‐way analyses of variance (ANOVA) was used for comparisons among multiple groups. Data are presented as means ± standard errors of the mean (SEMs). Statistical significance is defined as NS (non‐significant), *p* < 0.05, *p* < 0.01, *p* < 0.001. The statistical details of experiments are shown in the figure legends, including the statistical tests used, exact value of n, what n represents. All the statistical details of experiments can be found in the figure legends.

### 4.15 Ethic Approval

Participants in this study were recruited from the Parkinson's Disease and Movement Disorders Multicenter Database and Collaborative Network in China (PD‐MDCNC; http://pd‐mdcnc.com/). The study was approved (#202005124) by the Medical Ethics Committee of Xiangya Hospital, Central South University. The study was conducted in accordance with the Declaration of Helsinki and was registered with the Office of Human Genetic Resource Management. All participants provided written informed consent before enrollment and consented to the collection of peripheral blood samples for research purposes.

## Author Contributions

Z.G., B.T., Z.L., Y.Z., and P.L. initiated the project. H.G., and Z.L. collected samples from patients and conducted analysis. J.L., X.L., Y.S., and Y.Z. designed and synthesized all compounds. T.L. generated TMEM175 mutants and examined the lysosome expression pattern of TMEM175 mutants. S.L., R.C., and T.L. conducted lysosomal immunoprecipitation, LysoTrackerTM Red staining and generation of stable cell lines. Z.J., and Y.H. performed electrophysiological recordings and drug screening. Y.Z., and S.W. presented the channel structure with the software. M.H., and Y.Z. participated in site‐directed mutagenesis and figure preparation., Z.G., B.T., Z.L., Y.Z., and P.L. supervised the project and participated in manuscript editing., Z.L., Y.Z., and P.L. wrote the manuscript with input from all authors.

## Consent to Participate

Informed consent was obtained from all individual participants in the study.

## Consent for Publication

Patients signed informed consent for the publication of their data and photographs.

## Conflicts of Interest

The authors declare no conflict of interest.

## Supporting information




**Supporting File**: advs76738‐sup‐0001‐SuppMat.pdf.

## Data Availability

The data that support the findings of this study are available in the supplementary material of this article.
