# Supplementary material for: Trafficking Deficiency of TMEM175 Variants in Parkinson's Disease Pathogenesis and the Prospects of Precision Medicine
Source: Adv Sci (Weinh). 2026 Jul 31;13(57):e76738. doi: 10.1002/advs.76738 (PMC13426094; doi:10.1002/advs.76738)
Supplement: Supplementary file 1 [file ADVS-13-e76738-s001.pdf]

ADVANCED SCIENCE

Supporting Information

Trafficking deficiency of TMEM175 variants in Parkinson's disease pathogenesis and the prospects of precision medicine

Ting Luo[#], Yu He[#], Shuyao Li[#], Haoyu Guan[#], Ruili Cui[#], Yu Shi[#], Zhongwen Jiang, Siyu Wang, Xuan Li, Jiyuan Li, Mei Hu, Yupeng Zhou,Beisha Tang, Yanyan Zhang, Zhaobing Gao, Yu Zhou[*], Zhenhua Liu[*], Ping Li[*]

The PDF file includes:

Fig. S1 to S11

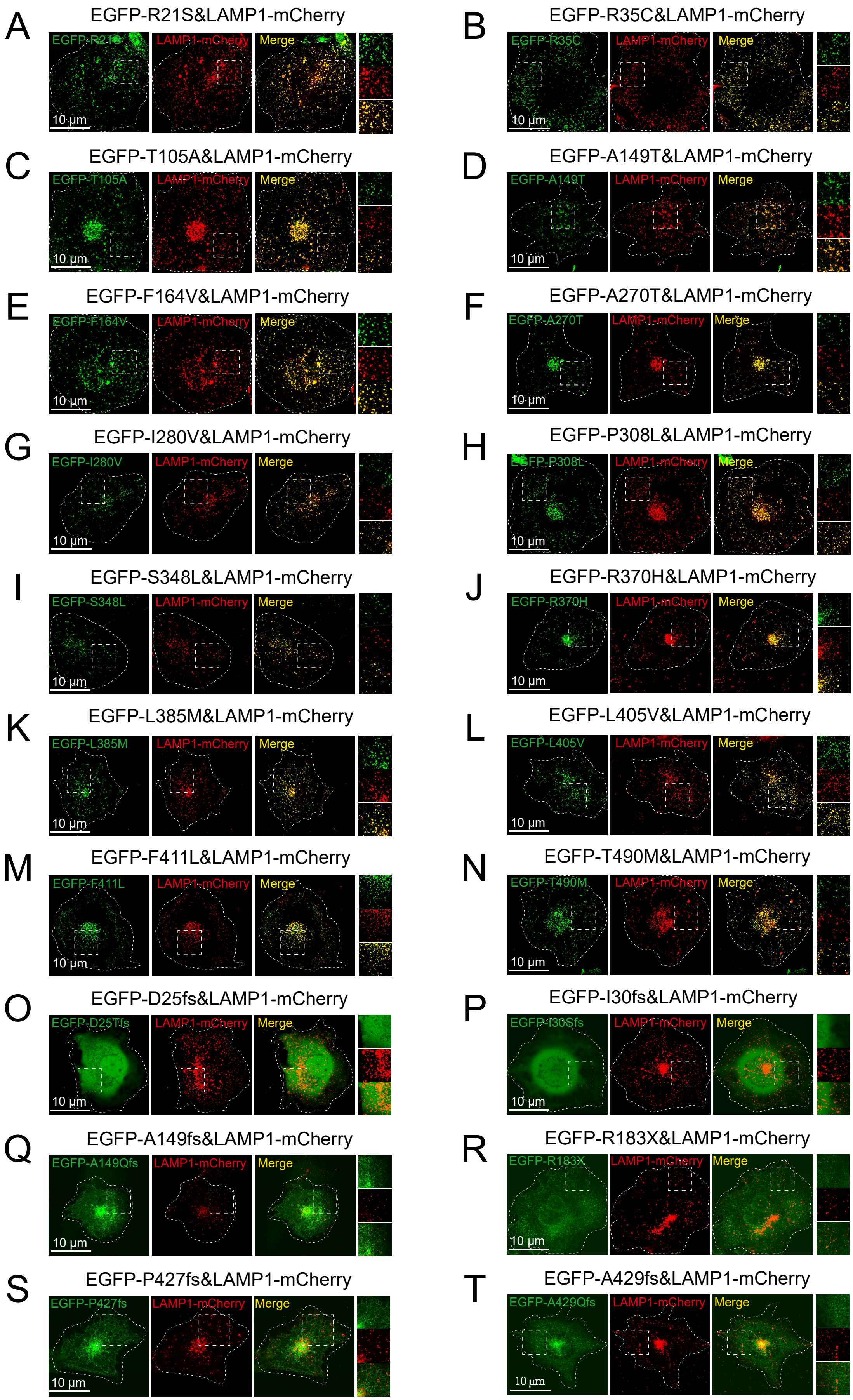

Luo et al., Fig. S1

Figure S1. The subcellular expression of 14 variants of TMEM175 associated with PD. (**A-M**) Representative images exhibit the lysosomal co-localization of EGFP-R21S (**A**), EGFP-R35C (**B**), EGFP-T105A (**C**), EGFP-A149T (**D**), EGFP-F164V (**E**), EGFP-A270T (**F**), EGFP-I280V (**G**), EGFP-P308L (**H**), EGFP-S348L (**I**), EGFP-R370H (**J**), EGFP-L385M (**K**), EGFP-L405V (**L**), EGFP-F411L (**M**), and EGFP-T490M (**N**) with LAMP1-mCherry in TMEM175 KO COS1 cells. Scale bar = 10 μm. (**O-T**) Representative images exhibit the lysosomal absence of EGFP-D25fs (**O**), EGFP-I30fs (**P**), EGFP-A149fs (**Q**), EGFP-R183X (**R**), EGFP-P427fs (**S**), and EGFP-A429fs (**T**) in TMEM175 KO COS1 cells. Scale bar = 10 μm. n=4.

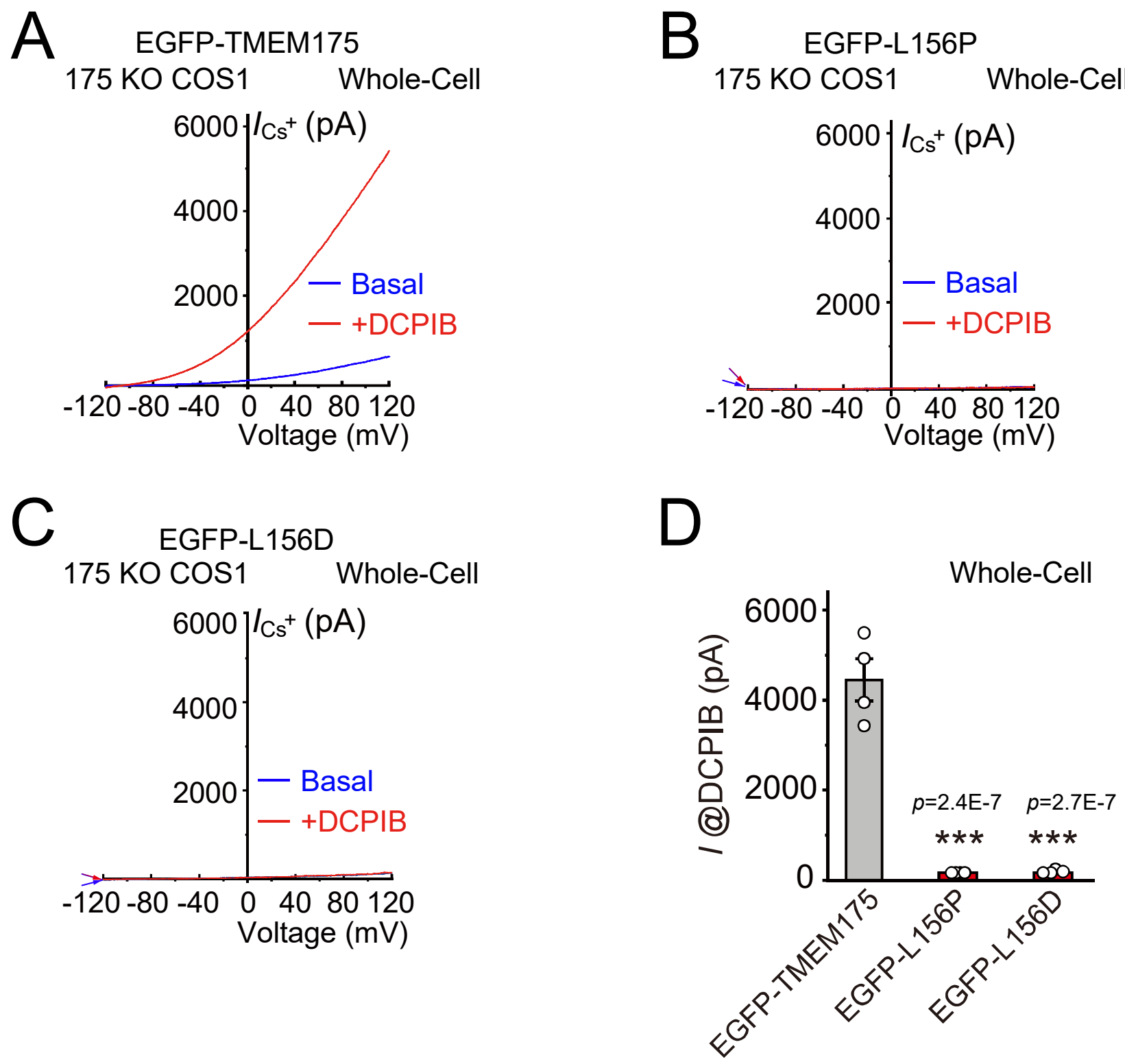

Luo et al., Fig. S2

Figure S2. EGFP-L156P and EGFP-L156D are absent on the plasma membrane. (**A**-**C**) Representative Cs^+ currents induced by DCPIB from the plasma membrane in TMEM175 KO COS1 cells overexpressed with EGFP-TMEM175 (**A**), EGFP-L156P (**B**), and EGFP-L156D (**C**), respectively. For the Cs^+ current, the pipette solution is (in mM): 145 Cs^+, 140 MSA^-, 5 Cl^-, 20 HEPES, pH_L=7.20, and the bath solution is: 145 $NMDG^+$, 140 MSA^-, 5 Cl^-, 20 HEPES, pH_C=7.20. A ramp protocol (-120 mV to +120 mV, 200 ms, holding at 0 mV) was used to record the current at 5s intervals. (**D**) Quantification of the increased fold of the current amplitude in the presence of DCPIB in experiments shown in (**A-C**). Data are mean ± s.e.m. from four independent experiments (n=4). One-way ANOVA.

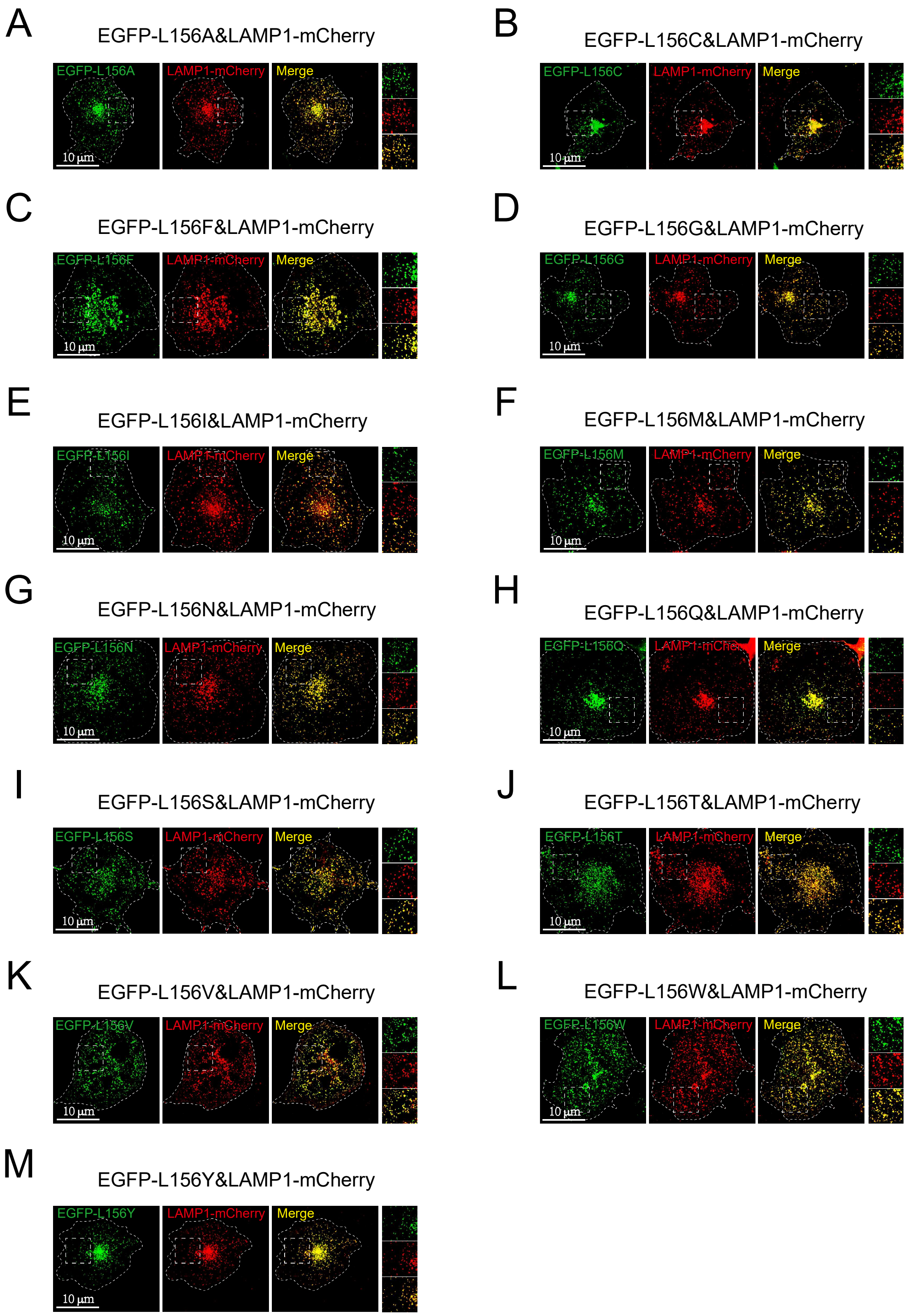

Luo et al., Fig. S3

Figure S3. The subcellular expression of 13 mutants of L156. (**A-M**) Representative images exhibit the lysosomal co-localization of EGFP-L156A (**A**), EGFP-L156C (**B**), EGFP-L156F (**C**), EGFP-L156G (**D**), EGFP-L156I (**E**), EGFP-L156M (**F**), EGFP-L156N (**G**), EGFP-L156Q (**H**), EGFP-L156S (**I**), EGFP-L156T (**J**), EGFP-L156V (**K**), EGFP-L156W (**L**), and EGFP-L156Y (**M**) with LAMP1-mCherry in TMEM175 KO COS1 cells. Scale bar = 10 μm. n=4.

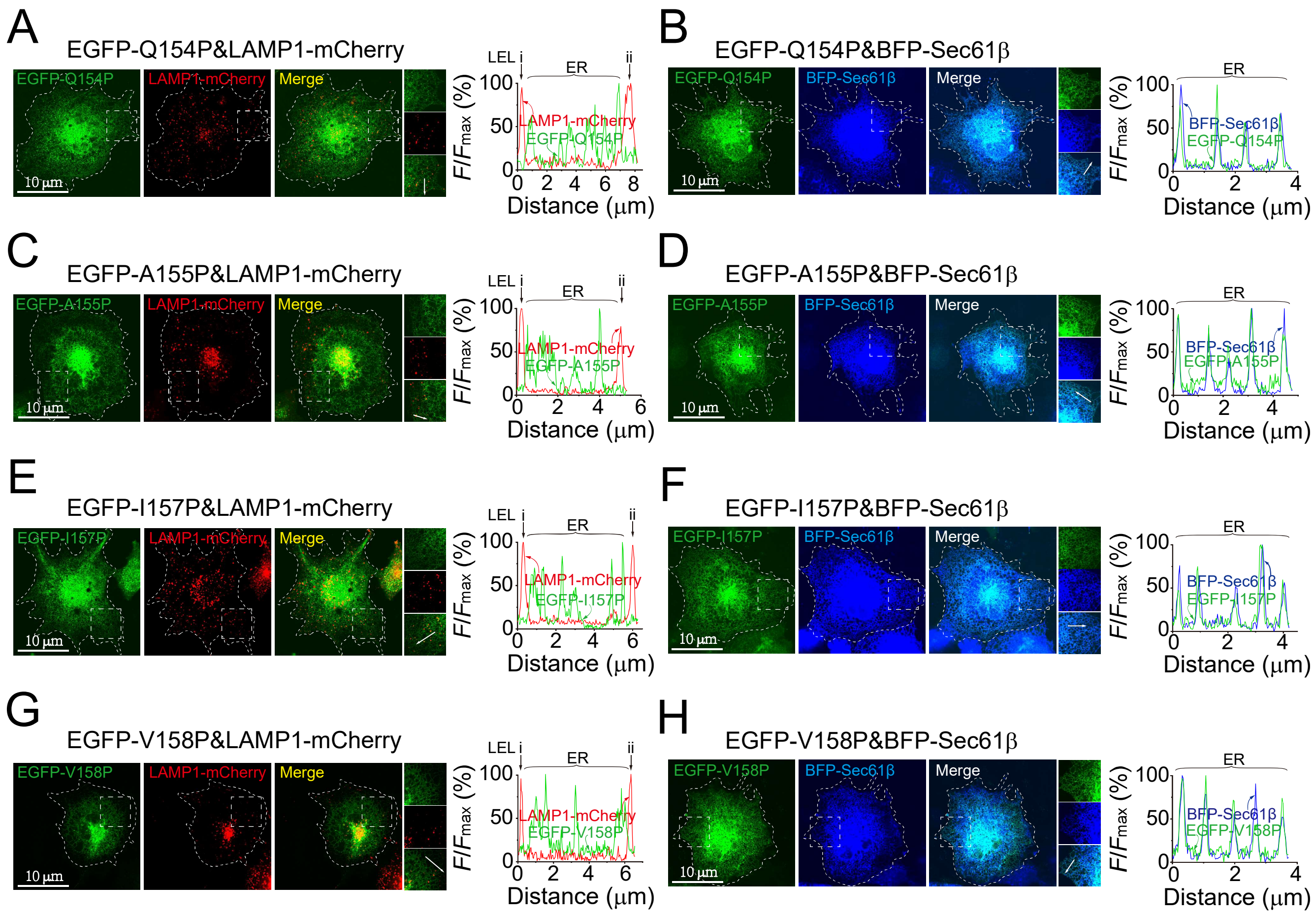

Luo et al., Fig. S4

Figure S4. The intracellular mis-location of 4 mutants of residues in TM4-1. (**A**) The absence of EGFP-Q154P in lysosomes. The graph to the *Right* of each group of images is a line scan through two separated lysosomes (LEL-i and LEL-ii) shown in the boxed region, indicating the intensity of EGFP-L391P (green lines) and LAMP1-mCherry (red lines) along the white arrow. Scale bar = 10 μm. n=4. (**B**) The presence of EGFP-Q154P in the ER. The graph to the *Right* of each panel shows the fluorescence intensity of a line scan (white arrows on the blown-up image) through the double-labeled object. Scale bar = 10 μm. n=4. (**C**) The absence of EGFP-A155P in lysosomes. (**D**) The presence of EGFP-A155P in the ER. n=4. (**E**) The absence of EGFP-I157P in lysosomes. n=4. (**F**) The presence of EGFP-I157P in the ER. n=4. (**G**) The absence of EGFP-V158P in lysosomes. n=4. (**H**) The presence of EGFP-V158P in the ER. n=4.

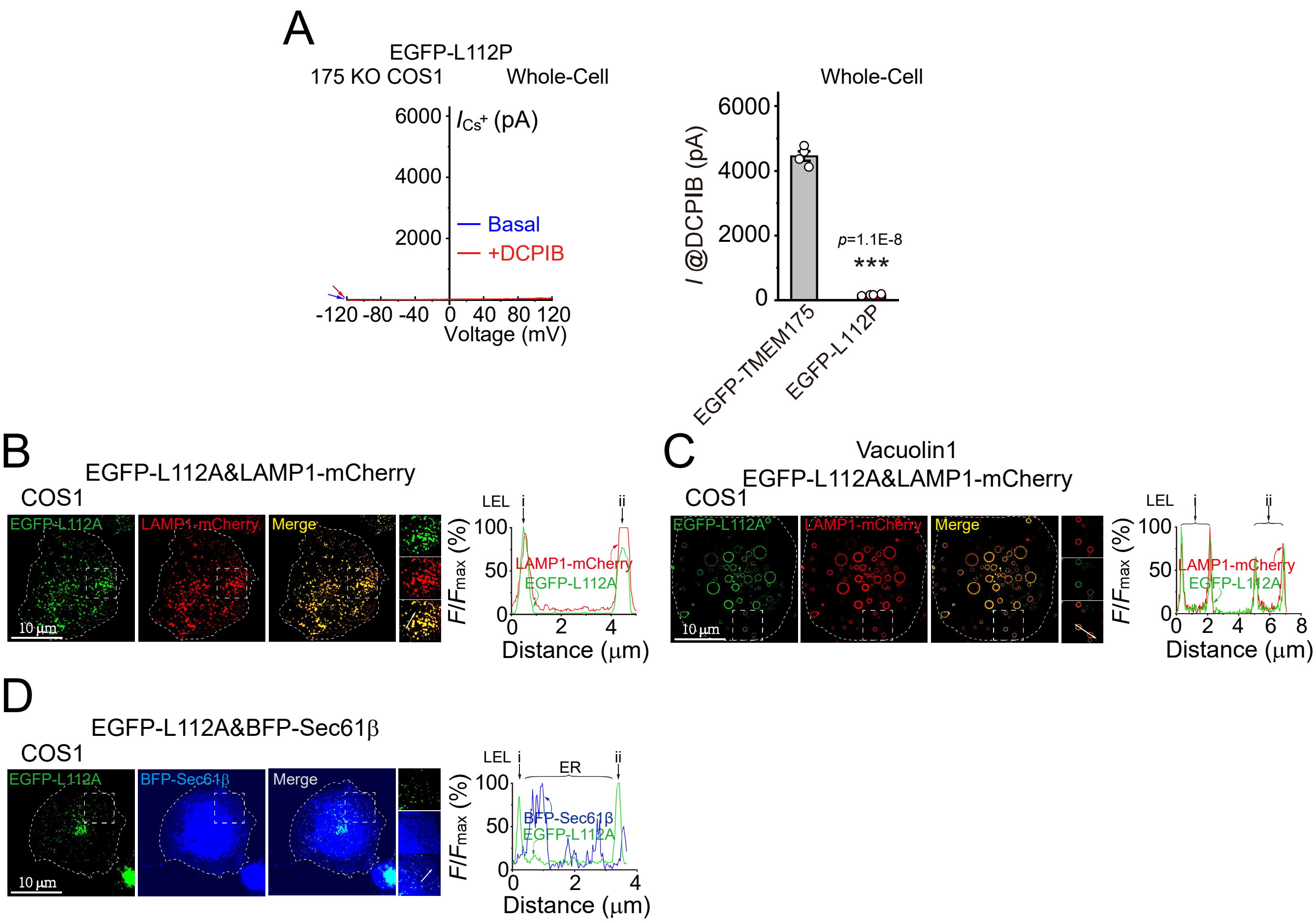

Luo et al., Fig. S5

Figure S5. EGFP-L112A remains expressed in lysosomes. (**A**) Representative *I-V* curves and the increased fold of the current amplitude induced by DCPIB from lysosome membranes in TMEM175 KO COS1 cells overexpressed with EGFP-L112P. Data are mean ± s.e.m. from four independent experiments (n=4). One-way ANOVA. (**B** and **C**) Representative images show the lysosomal expression of EGFP-L112A in TMEM175 KO COS1 cells in the absence (**B**) and presence of vacuolin-1 (**C**). The graph to the *Right* of each group of images is a line scan through two separated lysosomes (LEL-i and LEL-ii) shown in the boxed region, indicating the intensity of EGFP-L112A (green lines) and LAMP1-mCherry (red lines) along the white arrow. Cells were treated with vacuolin-1 for 12-18h after transfection. Scale bar = 10 μm. n=4. (**D**) The absence of EGFP-L112A in the ER. The graph to the *Right* of each panel shows the fluorescence intensity of a line scan (white arrows on the blown-up image) through the double-labeled object. Scale bar = 10 μm. n=4.

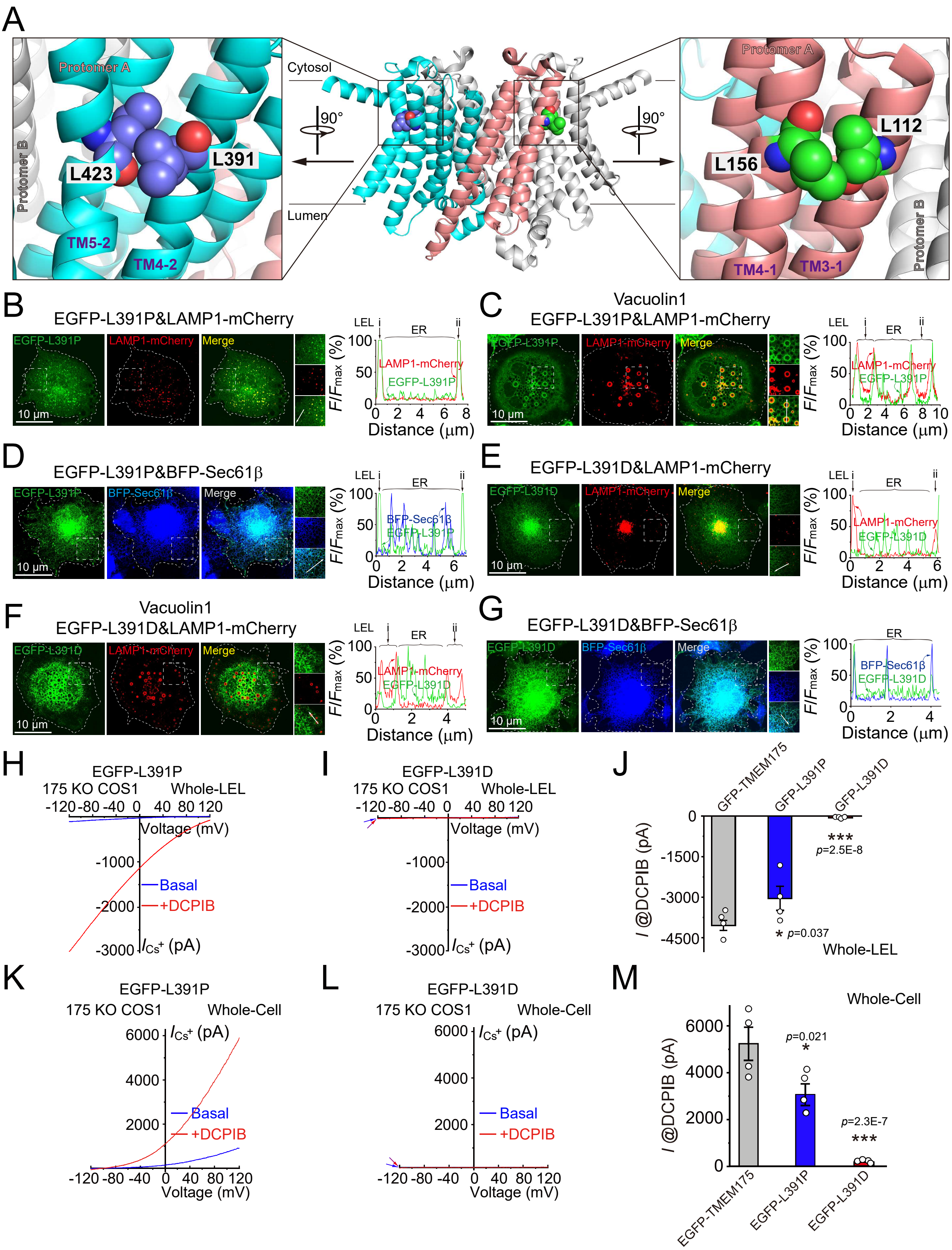

Luo et al., Fig. S6

Figure S6. The regulation of TM4-2 on the expression pattern of TMEM175. (**A**) The structural comparison of the interface between TM4-2 and TM5-2 in the repeat II (cyan) and the interface between TM3-1 and TM4-1 in the repeat I (deep salmon) of the TMEM175 channel (PDB:6WC9). Protomers A and B are shown in deep salmon plus cyan and grey, respectively. The residues are shown as spheres. (**B** and **C**) Representative images show the lysosomal localization of EGFP-L391P in TMEM175 KO COS1 cells in the absence (**B**) and presence of vacuolin-1 (**C**). The graph to the *Right* of each group of images is a line scan through two separated lysosomes (LEL-i and LEL-ii) shown in the boxed region, indicating the intensity of EGFP-L391P (green lines) and LAMP1-mCherry (red lines) along the white arrow. Cells were treated with vacuolin-1 for 12-18h after transfection. Scale bar = 10 μm. n=4. (**D**) Representative images show the co-localization of EGFP-L391P with BFP-Sec61β in TMEM175 KO COS1 cells. Scale bar = 10 μm. n=4. (**E** and **F**) Representative images show the absence of the co-localization of EGFP-L391D with LAMP1-mCherry in TMEM175 KO COS1 cells in the absence (**E**) and the presence of vacuolin-1 (**F**). Scale bar = 10 μm. n=4. (**G**) Representative images show the co-localization of EGFP-L391D with BFP-Sec61β in TMEM175 KO COS1 cells. Scale bar = 10 μm. n=4. (**H** and **I**) Representative *I-V* plots of DCPIB-induced currents from the lysosome in TMEM175 KO cells transfected with EGFP-L391P (**H**) and EGFP-L391D (**I**). (**J**) DCPIB activated currents from cells transfected with EGFP-L391P but not EGP-L391D. Data are mean ± s.e.m. from four independent experiments (n=4). One-way ANOVA. (**K** and **L**) Representative images show the co-localization of EGFP-L423P with LAMP1-mCherry in TMEM175 KO COS1 cells in the absence (**K**) and presence of vacuolin-1 (**L**). Scale bar = 10 μm. (**M**) Representative images show the co-localization of EGFP-L423P with BFP-Sec61β in TMEM175 KO COS1 cells. Scale bar = 10 μm.

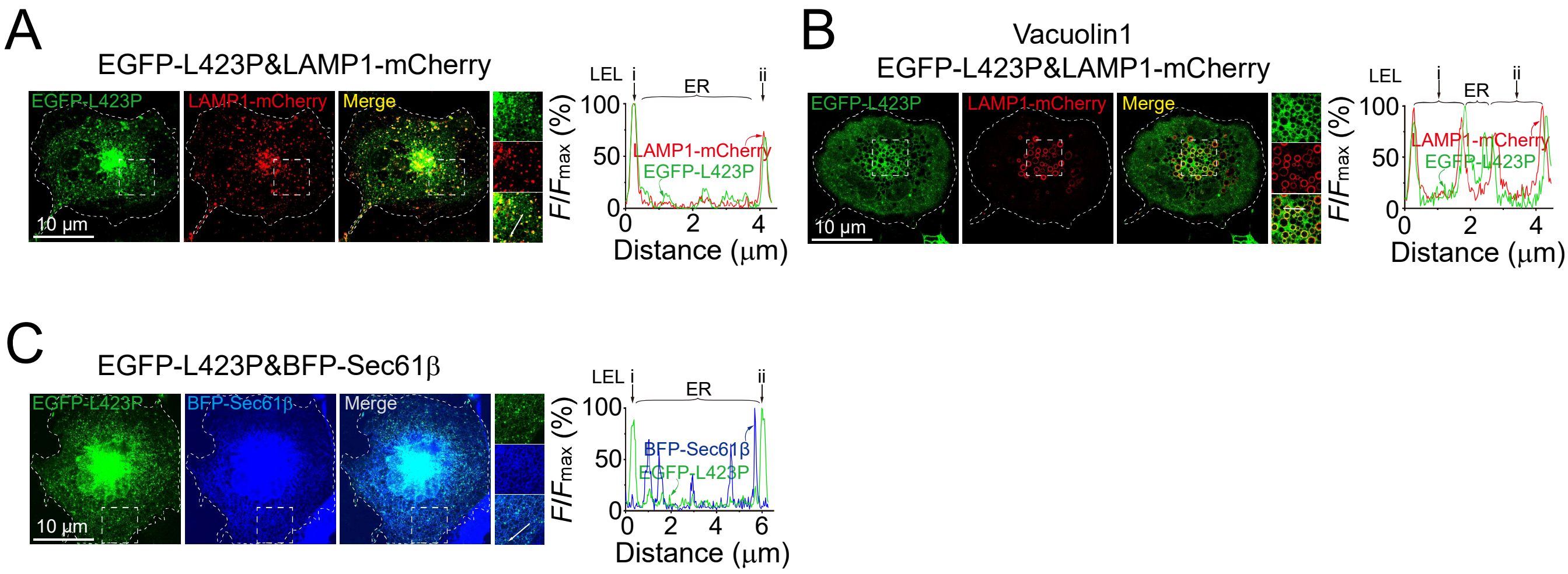

Luo et al., Fig. S7

Figure S7. EGFP-L423P is expressed in both the lysosome and ER. (**A** and **B**) Representative images show the absence of the co-localization of EGFP-L423P with LAMP1-mCherry in TMEM175 KO COS1 cells in the absence (**E**) and the presence of vacuolin-1 (**F**). Scale bar = 10 μm. n=4. (**C**) Representative images show the co-localization of EGFP-L423P with BFP-Sec61β in TMEM175 KO COS1 cells. Scale bar = 10 μm. n=4.

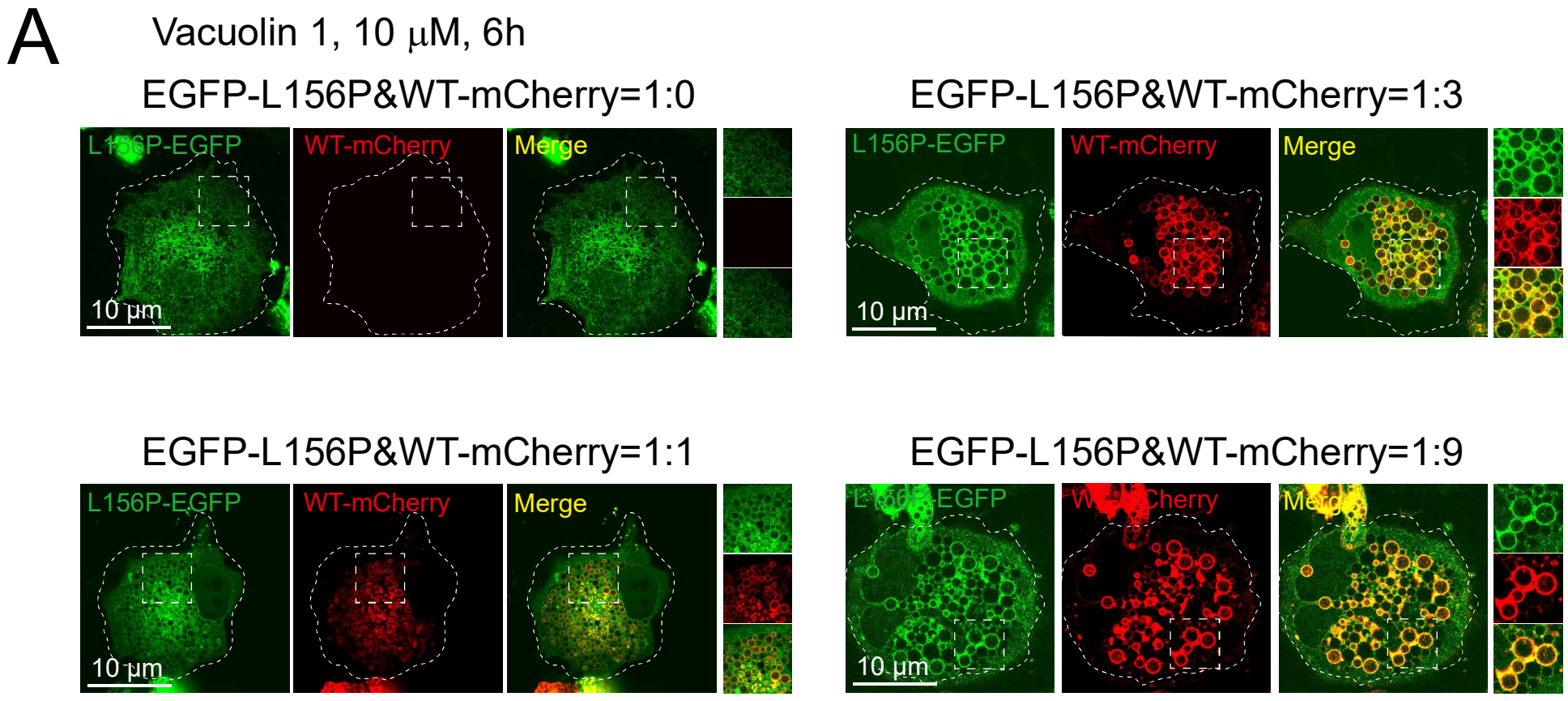

Luo et al., Fig. S8

Figure S8. The lysosomal expression of EGFP-L156P in the co-transfection with WT TMEM175-mCherry. (**A**) Representative images show the lysosomal expression of EGFP-L156P in TMEM175 KO COS1 cells after treatment with vacuolin-1 in the presence of WT TMEM175-mCherry in various ratios. Cells were treated with vacuolin-1 for 12-18h after transfection. Scale bar = 10 μm. n=4.

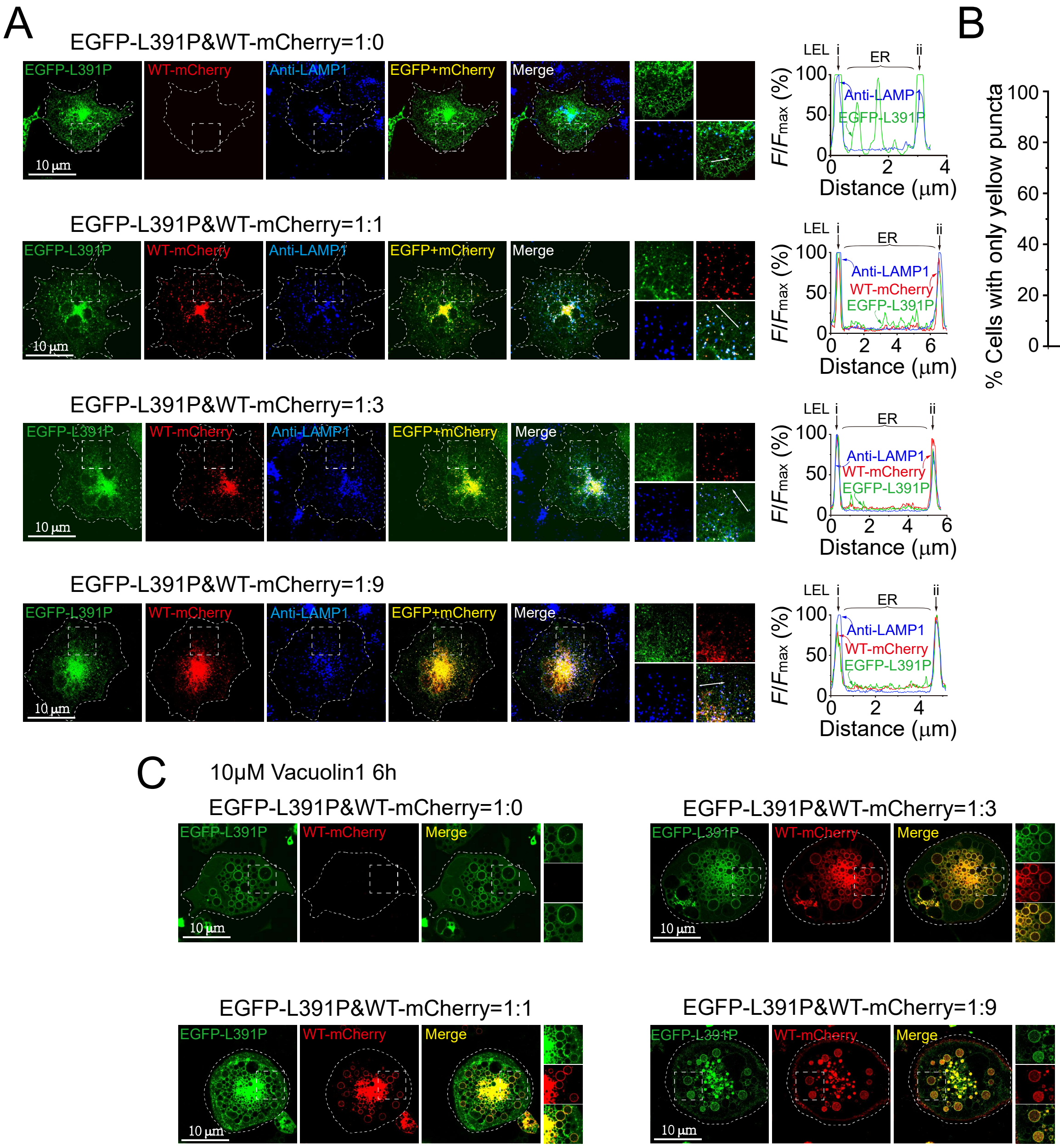

Luo et al., Fig. S9

Figure S9. The restoration of the lysosomal expression of EGFP-L391P by WT TMEM175-mCherry. (**A**) Representative images show the subcellular localization of EGFP-L391P in TMEM175 KO COS1 cells co-transfected with WT TMEM175-mCherry in various ratios. The graph to the *Right* of each group of images is a line scan through two separated lysosomes (LEL-i and LEL-ii) shown in the boxed region indicating the intensity of EGFP-L391P (green lines), WT-mCherry (red lines), and LAMP1 (blue lines) along the white arrow. Lysosomes were detected by immunostaining against LAMP1. Scale bar = 10 μm. (**B**) Quantification of percentage cells with the lysosomal expression of EGFP-L391P in experiments shown in (**A**). Data are mean ± s.e.m. from six independent experiments (n=6). 200-300 cells were selected for the analysis. One-way ANOVA. (**C**) Representative images show the lysosomal expression of EGFP-L391P in TMEM175 KO COS1 cells after treatment with vacuolin-1 in the presence of WT TMEM175-mCherry in various ratios. Cells were treated with vacuolin-1 for 12-18h after transfection. Scale bar = 10 μm. n=4.

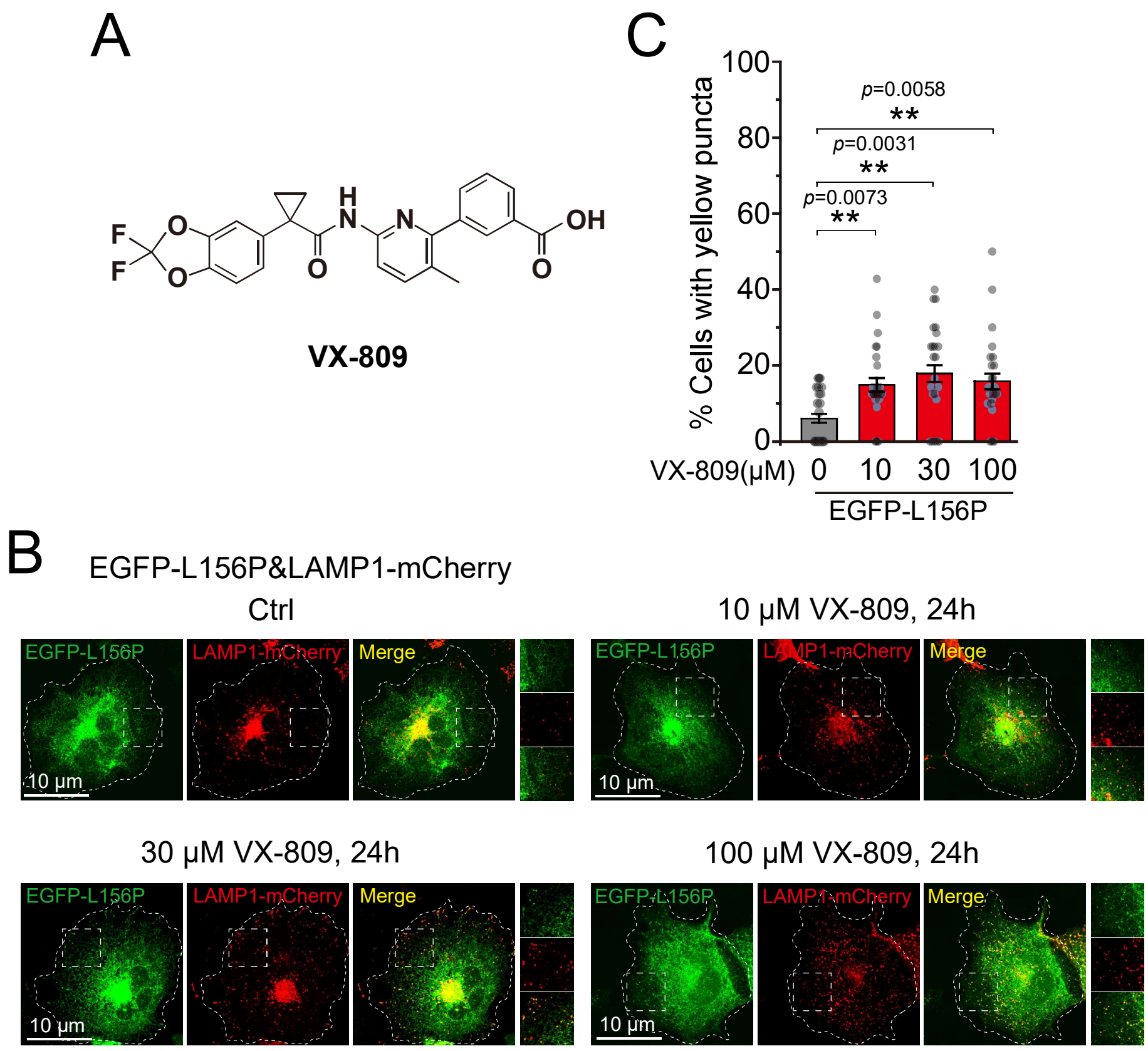

Luo et al., Fig. S10

Figure S10. VX-809 rescues the lysosomal localization of EGFP-L156P. (**A**) Chemical structure of VX-809. (**B**) Representative images show the lysosomal co-localization of EGFP-L156P with LAMP1-mCherry in TMEM175 KO COS1 cells after treatment with VX-809. Scale bar = 10 µm. (**C**) Quantification of percentage cells with the lysosomal expression of EGFP-L156P in experiments shown in (**B**). Data are mean ± s.e.m. from six independent experiments (n=6). 200-300 cells were selected for the analysis. One-way ANOVA.

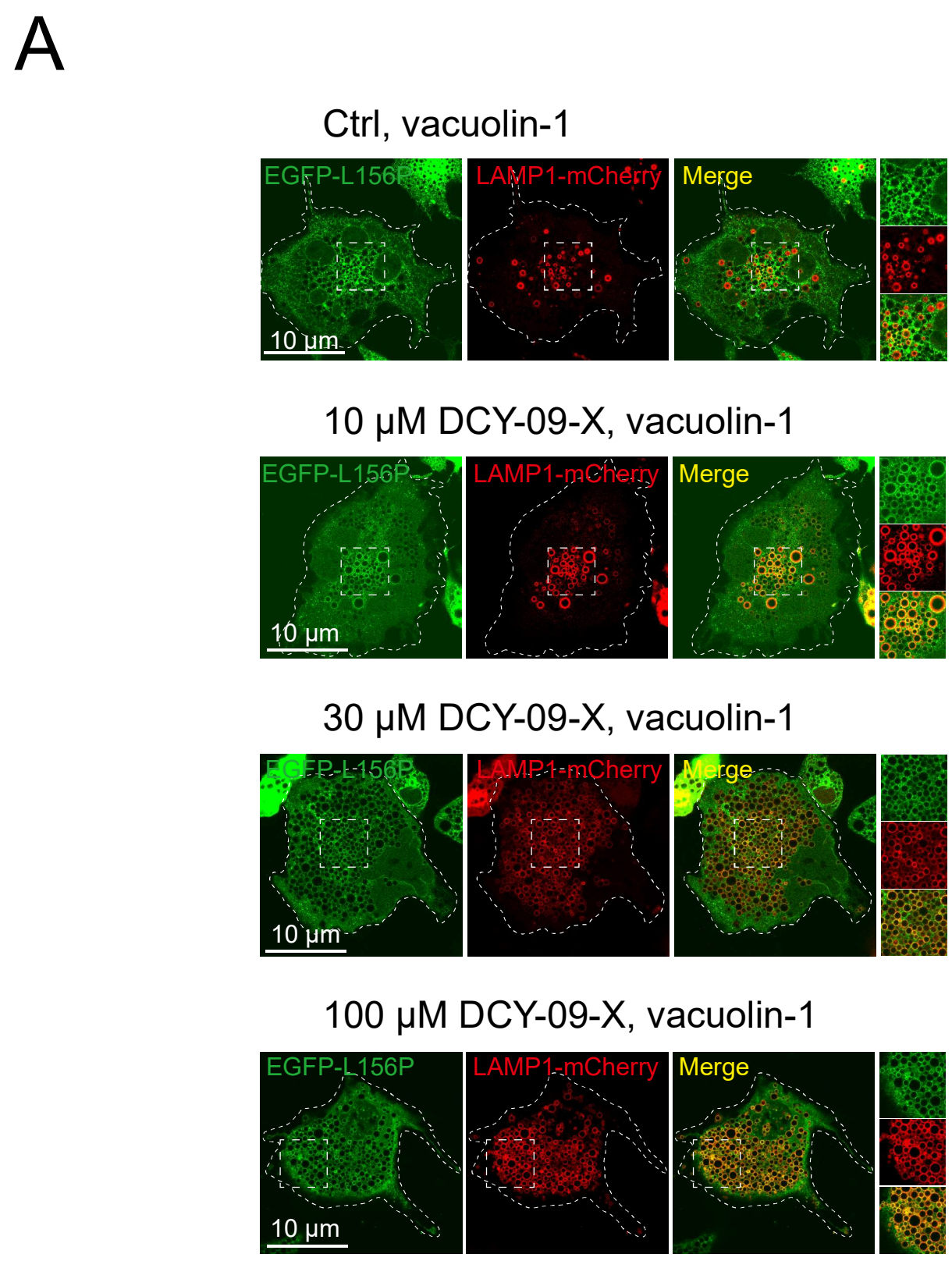

Luo et al., Fig. S11

Figure S11. DCY-09-X rescues the lysosomal localization of EGFP-L156P in the enlarged lysosomes. (**A**) Representative images show the lysosomal localization of EGFP-L156P in TMEM175 KO COS1 cells in the application vacuolin-1 after treatment with DCY-09-X. Cells were treated with vacuolin-1 for 12-18h after transfection. Scale bar = 10 μm. n=4.
